# Genomics-Enabled Next-Generation Breeding Approaches for Developing System-Specific Drought Tolerant Hybrids in Maize

**DOI:** 10.3389/fpls.2018.00361

**Published:** 2018-04-11

**Authors:** Thirunavukkarsau Nepolean, Jyoti Kaul, Ganapati Mukri, Shikha Mittal

**Affiliations:** Maize Research Lab, Division of Genetics, ICAR-Indian Agricultural Research Institute, New Delhi, India

**Keywords:** accelerated line breeding, big data, breeding informatics, drought, genomics, maize, next-generation breeding, rainfed

## Abstract

Breeding science has immensely contributed to the global food security. Several varieties and hybrids in different food crops including maize have been released through conventional breeding. The ever growing population, decreasing agricultural land, lowering water table, changing climate, and other variables pose tremendous challenge to the researchers to improve the production and productivity of food crops. Drought is one of the major problems to sustain and improve the productivity of food crops including maize in tropical and subtropical production systems. With advent of novel genomics and breeding tools, the way of doing breeding has been tremendously changed in the last two decades. Drought tolerance is a combination of several component traits with a quantitative mode of inheritance. Rapid DNA and RNA sequencing tools and high-throughput SNP genotyping techniques, trait mapping, functional characterization, genomic selection, rapid generation advancement, and other tools are now available to understand the genetics of drought tolerance and to accelerate the breeding cycle. Informatics play complementary role by managing the big-data generated from the large-scale genomics and breeding experiments. Genome editing is the latest technique to alter specific genes to improve the trait expression. Integration of novel genomics, next-generation breeding, and informatics tools will accelerate the stress breeding process and increase the genetic gain under different production systems.

## Introduction

Increasing the food production is a challenge to feed the global population that is expected to reach about 9 billion by 2050. Maize (*Zea mays* L.) is one of the important crops and its acreage is increasing considerably especially in Asia and Africa. The world productivity of maize was 5.5 ton/ha whereas in developing economies it is about 2.5 ton/ha (https://apps.fas.usda.gov/psdonline/circulars/production.pdf). The Intergovernmental Panel on Climate Change (IPCC) prediction says that the mean temperatures around the planet may rise between 2 and 5°C or more by 2050 (www.ipcc.ch). Among other hurdles, the changes in the climate followed by its consequences are the major threats to different crop production systems. The tropical and subtropical climates occupying 160 million hectares are most vulnerable, since climate changes, adversely affect the dynamics of temperature and water availability. And it is also important to note that most of the thickly populated and developing countries are located in these climates, any adversity in agriculture production will cause greater damage to the food security of millions of people. The common production system in tropical and subtropical climates is rain-fed (Edmeades, 2008) and the global yield loss is nearly 25% in the rain-fed system. About 19 million hectares of the tropical Asia is rain-fed of which 80% is drought-prone. More losses are expected in the tropical system owing to the unpredictable rainfall condition (Mhike et al., 2012).

From the “Mendelian era” of nineteenth century we have now reached the stage of “Genomics era” in twenty-first century where several new tools and techniques are available to understand the genetics of traits and accelerate the breeding process. The growth of “genomics” has become rapid in the last two decades. Sequencing of DNA and RNA are no longer the constraints and millions of SNPs can be generated in no time with the help of modern sequencers. Identification and understanding the function of genes are possible with the help of *omics* which include genome, transcriptome, and proteome and so on. The large-scale data from such genome-scale studies also ushered in new branches in bio-informatics called big-data management.

At the same time, new “breeding techniques” have rapidly emerged to reduce the breeding cycles and improve the genetic gain. Linkage and linkage-disequilibrium based QTL mapping approaches were revolutionized the way of understanding the genetics of traits. Introgressing QTLs into the target genotypes through marker-assisted breeding has improved several traits. New line breeding and whole-genome based selection approaches such as genomic selection (GS) has come up to further accelerate the breeding cycle and improve the genetic gain in the breeding programme. Currently genome editing techniques are available to develop customized genotypes.

Results from various genomics and novel breeding experiments on drought tolerance in maize have started coming up in the public domain. The review has compiled those experiments as well as explained the strategies and opportunities to breed drought tolerant hybrids for different maize production systems.

## Production system-based stress breeding

Breeding for drought tolerance specific to the target production systems would provide more dividends since the systems decide the type of traits to be phenotyped to breed effective maize hybrids and to maximize the genetic gain. Drought stress is predominant in the low input rain-fed system which occupies more than 70% of the maize production systems in the developing world. Several component traits that belong to morpho-physiological categories include seedling vigor, root traits (volume, deepness, spread, primary, and secondary structures, hairs), leaf traits (hair, rolling, chlorophyll, delayed senescence), synchronized male-female flowering, stomatal regulation, evapo-transpiration, relative-water content, canopy temperature, hormones, osmotic adjustment, anti-oxidants, enzymes, etc. have been proposed to understand and improve the drought tolerance in maize. In the rain-fed system of sub-tropical regions, the seeds are sown after the first shower of the rain. Early emergence and seedling vigor are the important traits so the plants could emerge fast and reach to the vegetative stage. The next critical stage is flowering where male and female flowering should be synchronized for the effective pollination. Hence, breeding for reducing anthesis and silking interval (ASI) assumes great significance under rain-fed system (Araus et al., 2012). Positive correlation between ASI and grain yield in maize has been reported previously (Monneveux et al., 2006). During the seed setting stage, the plant survives with the existing soil moisture and maximizes its grain filling efficiency. Green leaves with delayed senescence (stay green) retain the moisture and help better in photosynthesis during grain filling stage (Lee and Tollenaar, 2007). Breeding genotypes with the ability to convert the source to the sink rapidly is another preferable approach, so effective grain filling is possible in shorter duration. Since efficient mining of moisture is vital for survival and reproduction, a better deep root system will be supportive throughout the plant life cycle in rain-fed condition. Breeding medium maturity hybrids by considering the above-mentioned traits is necessary to sustain the productivity.

Maize is also cultivated in high-input irrigated system with assured resources and clear-cut management. The use-efficiency of irrigation water is often low and around 50% of the increase in demand for water could be saved by increasing the effectiveness of irrigation (Seckler et al., 1998). What is necessary in the irrigated system is to reduce the quantity of water per irrigation or to reduce the number of irrigations per crop cycle with optimized water-use-efficiency. The aerial parts of the plant play an important role in deciding water-requirement of the plant. Plant types with better osmotic adjustments (OA) and less evapo-transpiration rate are the efficient ones under this condition. Genotypes that can save the water at least to the tune of 10–20% will greatly reflect in the environmental sustainability. The efforts in reducing the water consumption and improving the environmental sustainability in the irrigated system will also lead to the reduction of carbon foot prints.

On the other hand, it is very difficult to differentiate micro traits such as hormone levels, enzymes, signaling molecules, ROS scavenging mechanisms, etc. and their possible interactions unique to the production systems. Since many drought-associated traits are interrelated across systems, selecting system-specific macro traits by keeping other micro traits in common would be a plausible strategy. Additionally, it is imperative to understand the correlation between drought tolerant genes with grain yield components. While breeding for drought tolerance, caution should be taken since the tolerant genes operating in different pathways may lead to yield penalty. Breaking the negative association between tolerant genes and yield components, if any, is an important strategy to sustain the grain yield in any production system.

## Utilization of maize germplasm

Over 50,000 global maize accessions conserved at several gene banks (Hoisington et al., 1999) including 28,000 accessions in CIMMYT (http://www.cimmyt.org/germplasm-bank/) explain the quantum of genetic variability available in maize germplasm. The artificial selection over the period of time caused genetic drift and reduced the allelic diversity in the elite gene pool (Xu et al., 2009). It has to be increased by incorporating exotic wild germplasm and landraces into the elite germplasm pool to tape-out the new genes including the stress tolerant genes. Mining novel alleles for drought tolerance from the unutilized germplasm is possible since only 5% of the germplasm is globally used in commercial breeding (Hoisington et al., 1999). Core sets in maize have been developed from different kinds of germplasm to capture the maximum allelic diversity with minimum number of genotypes (Wang et al., 2008; Wen et al., 2012).

Trait-specific core set such as “drought-core” is necessary to exploit the genetic variability that exists in the wild and cultivated germplasm. Phenotyping a large set of genetically diverse maize germplasm under drought stress would be helpful to understand the variability as well as to develop a core set. The “drought-core” could provide an opportunity to identify novel genes using genomic approaches. Additionally, drought tolerant populations in maize have been developed using drought tolerance with good combining ability. Similarly, drought-specific pools have been developed using ASI, leaf senescence, and leaf rolling traits (Edmeades et al., 1999; Monneveux et al., 2006). Such pools are useful for enriching the drought tolerant alleles and genotypes extracted from the pool could be for further used in stress breeding programmes.

## Accelerated line breeding

Development of potential parental lines, which is a vital component of a maize breeding programme, is challenging and time consuming process. Through conventional pedigree and bulk methods, 7–8 generations are required to get complete homozygous lines from heterozygous founder stocks. Development of homozygous lines through accelerated line breeding (ALB) approaches is expected to save resources as well as speed-up the product delivery.

Doubled-haploid (DH) production has become a routine technology in maize genetics and breeding (Rober et al., 2005; Geiger and Gordillo, 2009). It is credited with significantly shortening of the breeding cycle by development of completely homozygous lines in two generations as well as simplifying logistics (Geiger and Gordillo, 2009), including requirement of less time, labor, and financial resources; the time and resources thus saved could be potentially channelized for implementing more effective selections and for accelerated release of elite cultivars. Tested genetic stocks are now used for rapid development of homozygous lines. Using Haploid inducers (HI) in maize, only two generations are required to generate homozygous lines (De La Fuente et al., 2013). Earlier, genetic stock 6 was used to produce haploids in maize (Coe, 1959). New generation inducer lines derived from genetic stock 6 with higher induction rates are now available. RWS is one of such inducing lines (Rober et al., 2005) and its sister line RWS-76 (Geiger and Gordillo, 2009) has a haploid induction rate of 8–10% in tropical maize (Prigge et al., 2012). DH technology in maize has become a huge success both in public and private sectors (De La Fuente et al., 2013). The haploid inducers are useful in developing homozygous inbreds from heterozygous and heterogeneous populations, converting male fertile lines into cytoplasmic sterile lines, development of homozygous QTL mapping populations, development of trait mapping panels from landraces, marker-assisted backcross breeding (MABC) and, development of genetic stocks such as isogenic lines and segmental substitution lines.

Rapid generation advancement (RGA) is a new technique to reduce the life cycle of the plant so that inbreds can be rapidly obtained. Unlike DH where lethal alleles also fixed in the population, the population developed from RGS has less likely to contain recessive alleles due to natural and artificial selection. Similar to recombinant inbred line (RIL) development procedure, more recombinant events are allowed in RGA technique. The principle behind the technique is that many generations per year could be obtained by following different strategies. For example, by reducing the life cycle of a full season maize that matures in 100 to 110 days by 30–50%, may enable the breeders to take many crops per year thereby accelerating the line breeding process. In RGA, strategies such as seed treatment, nutrient management, application of hormones, accelerated-flowering, temperature control, breaking seed dormancy, embryo rescue, and combinations thereof are involved in reducing the life cycle. RGA has been attempted in several crops to shorten the breeding cycle (Chickpea-Gaur et al., 2007; Sorghum-Rizal et al., 2014; Rice-Tanaka et al., 2016). Once the RGA technique in maize is standardized, it could play a major role in hybrid breeding programme as well as in rapid generation of genetic stocks for genomics studies.

## Precision phenotyping

Phenotyping is an integral part of the drought breeding that contributes in understanding the genetics of drought tolerance and product development. The target traits need to be measured rapidly and precisely. Since many component traits of drought tolerance are controlled quantitatively, therefore improving the accuracy of phenotyping has acquired much attention to improve the heritability of the traits. Selection of primary and secondary traits (Monneveux et al., 2008) is the way to achieve drought tolerance in maize. Agronomically important traits such as grain yield and yield contributing traits are the primary traits considered for direct selection. ASI, root architecture and stay green are the important secondary traits to impart drought tolerance and contribute indirectly to yield (Nepolean et al., 2013). Hormones, free-radical scavengers, signaling molecules, enzymes, osmotic adjustment, leaf water potential (Thirunavukkarasu et al., 2014) are the molecular and physiological traits to be included in the selection process. Though the traits are classified in different categories, the agro-morpho-physiological traits should complement to each other for better productivity. Since the yield traits are the manifestation of several secondary traits, identification and selection of traits that are highly heritable, amenable for HTP phenotyping and positively correlated with yield traits is the key issue to achieve target level of drought tolerance (Maazou et al., 2016).

Precision phenotyping of direct and indirect traits is a challenging task in drought breeding programmes. Plant architecture including primary and secondary traits under drought stress could precisely be phenotyped in controlled conditions through newly emerged high-throughput and non-destructive techniques. Imaging techniques are widely used to phenotype the traits of maize. These have been used to phenotype the whole or specific part/s of the plant. Visible light imaging for whole part of the plant (Grift et al., 2011; Nagel et al., 2012), thermal imaging for whole shoot or leaf tissues (Araus et al., 2012), near infrared imaging for whole part of the plant (Spielbauer et al., 2009; Cook et al., 2012) and 3D imaging for shoot (Klose et al., 2009) have been reported in maize. The “phenotyping under controlled conditions” is helpful in large-scale phenotyping including trait mapping experiments. However, caution needs to be taken while deciphering the solution for drought tolerance since the controlled environment might not mimic the actual field condition as well as be less useful to study the genotype × environment interactions which are very crucial to understand the drought tolerance mechanisms. Alternatively, dynamic phenotyping in the controlled condition could be developed to reflect the actual field conditions. In the dynamic phenotyping the weather parameters are not static but variable all-through the plant life cycle akin to the target production systems.

Remotely-controlled unmanned aerial vehicles (UAVs) with appropriate instruments are also used to phenotype under open-field conditions. UAVs fly on the field and measure the target traits throughout the cropping period. Aerial phenotyping in maize using thermal images provide the normalized difference vegetation index (NDVI) and RGB data which can be further useful to measure a series of traits (Frank et al., 2015; Zaman-Allah et al., 2015).

## Maize genomic resources

**Figure 1:**
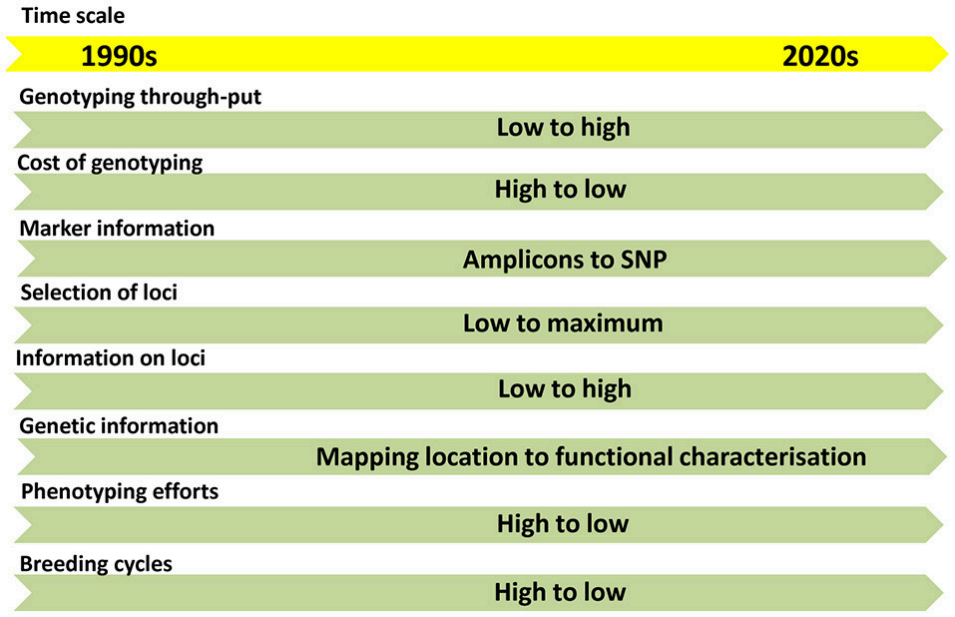
The trends in flow of information in genomics and molecular breeding in the last two decades.

With the advent of next-generation sequencing (NGS) technologies, genotyping is moving from amplicon-based low-throughput (LTP) to SNP-based high-throughput (HTP) systems (Figure 1). The abundance and cost-effective assays made SNP as the preferred marker choice for the genomic studies. The throughput of the SNPs can be modulated based on the purpose of genotyping. High-density SNPs are needed for high resolution fingerprinting, genome-wide association mapping (GWAS) and genomic selection (GS). Low to medium density SNPs are needed for genetic diversity analysis, QTL/trait mapping, marker-assisted selection (MAS), marker-assisted recurrent selection (MARS) and candidate gene-based selections.

Till recent past, NGS-based sequencing technologies were used to capture the SNPs at whole genome level. Now, third-generation sequencing technologies (Jiao and Schneeberger, 2017; Lee et al., in review) have emerged with a capability of generating long-read sequences. Pacific Biosciences (PacBio) Single Molecule Real Time (SMRT) sequencing, the Illumina Tru-seq Synthetic Long-Read technology and the Oxford Nanopore Technologies sequencing platform offer third-generation chemistries to capture the SNPs. The draft genome of the elite maize inbred line “Ph207” has been recently developed through Illumina Tru-seq Synthetic Long-Read technology (Hirsch et al., 2016).

Whole-genome sequencing will comprehensively reveal the structural architecture of a genome. Genome assemblies of 10 maize lines are available including reference lines B73, W22 and Mo17 (www.maizegdb.org). Close to 40,000 protein coding genes have been identified using B73 genome. The whole-genome sequencing also provides functional information on genes and SNPs. A total of 6,385,011 SNPs from 15 maize inbreds was identified by aligning the respective sequences against the maize reference B73 genome (Xu et al., 2014). Genome-wide SNPs will be eventually used in identification of haplotypes and in genetic mapping. The first generation haplotype map in maize called Hapmap1 was developed in 2009 (Gore et al., 2009) followed by second generation HapMap2 in 2012 (Chia et al., 2012). About 55 million SNPs have been identified in the HapMap2 set which comprises 103 lines from pre-domesticated and domesticated maize varieties. Now, the third generation Hapmap3 is available with the size of 3.83 million SNPs and InDels identified based on 1,218 maize germplasms (Bukowski et al., 2015).

Among restriction enzyme (RE)-based SNP identification, genotype-by-sequencing (GBS) (Elshire et al., 2011) has evolved as a cost-effective HTP method. Identification and utilization of SNPs through GBS have been performed in maize for various purposes. More than 100 SNPs were identified for root traits under drought stress condition from 955,690 SNPs generated though GBS (Zaidi et al., 2016). Genomic prediction of SNPs developed through GBS was studied from a diverse panel of 296 maize inbreds and 504 DH lines (Crossa et al., 2013) by phenotyping in controlled water-stress condition. GBS data has been used in identification of QTLs and SNPs in the maize nested association mapping (NAM) population for ASI and other drought-related traits under water-stress condition (Li et al., 2016). Through GBS, a total 383,145 putative SNPs have been identified from 21 diverse inbreds (7 flints and 14 dents) to assess the biomass production in maize (Muraya et al., 2015) and 261,055 SNPs were detected in an association mapping panel consisting of 282 genotypes to map tar leaf spot in maize (Cao et al., 2017). Another RE-based low cost rapid SNP discovery is restriction site-associated DNA sequencing (RADseq) (Baird et al., 2008). Using this technique, a total of 14,384 polymorphic SNPs was identified based on 34 maize inbreds (Tamaki et al., 2016).

In 2009, Illumina developed a golden gate assay Illumina® 1536 SNP chip and later it developed a high-density Illumina® MaizeSNP50 Beadchip (Lu et al., 2009; Wu et al., 2014). Using Affymetrix® Axiom® platform, a very high density Maize Genotyping Array has been developed to a level of 600K SNPs (MaizeSNP600K) (Unterseer et al., 2014). A lesser density array was also recently developed from the same platform with a density of 55,229 SNPs which covers variants from both tropical and temperate germplasms (Xu et al., 2017). These SNP chips have been used in genetic characterization of maize inbreds (Lorenz and Hoegemeyer, 2013; Thirunavukkarasu et al., 2013; Tian et al., 2015) and in GWAS (Cook et al., 2012; Li et al., 2013; Thirunavukkarasu et al., 2014).

Pre-selected and validated SNPs are necessary in tracking genotypes in MAS, MARS and GS. KASP™ offers customisable genotyping assay to run selected SNPs in applied breeding programmes (Thompson et al., 2014). A set of 275 SNPs through KASP assay was used to improve the drought tolerance of maize population through MARS (Abdulmalik et al., 2017). Testcross genotypes were selected based on the GEBVs of 1,214 SNPs using KASP assay (Vivek et al., 2017). Array Tape™ by Douglas Scientific, the OpenArray system from Life Technologies and Dynamic Arrays™ from Fluidigm are the other flexible systems available for HTP genotyping of selected SNPs (Thompson et al., 2014).

## Trait mapping

Bi-parental populations such as F_2_-derived, RILs, near-isogenic lines (NILs), etc. which follow the principle of linkage are used for coarse and fine mapping of QTLs. QTLs for grain yield and its component traits, ASI, abscisic acid (ABA) were identified under water-stressed conditions using F_2_-derived and RIL populations (Lebreton et al., 1995; Agrama and Moussa, 1996; Ribaut et al., 1997; Tuberosa et al., 1998a,b; Frova et al., 1999; Guo et al., 2008; Messmer et al., 2009; Almeida et al., 2013). A meta-QTL approach was used to identify QTLs for grain yield under drought condition from the data of 18 bi-parental mapping populations (Semagn et al., 2013).

Multi-parents population such as GWAS panel are developed by exploiting the principle of linkage disequilibrium (LD) (Thirunavukkarasu et al., 2013). They help in reducing the time taken for population development and provide an opportunity to test more alleles. GWAS is similar to that of fine mapping approach since it has the ability to identify genes when genome-wide SNPs are used in genetically diverse genotypes. GWAS has been employed in maize to understand the inheritance of complex traits. Using a GWAS panel, gene *ZmVPP1*, encoding a vacuolar-type H^+^ pyrophosphatase, identified as the most significantly contributing gene to the drought tolerance along with 42 candidate genes by exposing the seedlings to drought stress (Wang et al., 2016). The GWAS results were validated through linkage mapping, expression assays and candidate gene-based mapping experiments to employ the results in the selection programmes. The QTLs identified on chromosome 2, 7, and 8 for tar leaf spot using an association mapping approach were validated through linkage mapping using DH populations (Cao et al., 2017). A combined linkage and association mapping were performed to validate the QTLs for plant height and ear height in maize (Li et al., 2016).

Unequal allele frequencies in the members of GWAS panel produce false-positives which is considered as a major limitation of GWAS approach. The NAM population (Yu et al., 2008) was proposed to overcome the limitations of linkage mapping and association mapping. A large population created by systematic crossing of a common line with many founder lines provides an opportunity to exploit both linkage and linkage-disequilibrium models to map QTLs. NAM population has been used to identify QTLs for flowering time (Buckler et al., 2009), southern leaf blight (Kump et al., 2011), northern leaf blight (Poland et al., 2011) and drought tolerance (Li et al., 2016). MAGIC population (Kover et al., 2009), a balanced multi-parent cross design has been proposed in maize with higher resolution, power and elevated minor allele frequency (Dell'Acqua et al., 2015) against NAM population. The current statistical models based on multi-parent population approaches still have the limitation of including minor alleles in the analysis. New models are needed to include minor alleles present among the genotypes to understand their role in trait expression.

## Functional characterization of drought tolerance

Characterizing downstream events of QTLs is important to understand the functional mechanisms of the QTLs. Post transcriptional and post-translational changes have to be looked upon to understand the gene regulation process. mRNAs can be captured by exposing the genotypes under specific drought stress condition and by comparing with the control and/or sensitive genotypes. The differentially expressed genes (DEGs) will be functionally classified and annotated to realize their role in drought tolerance.

RNA sequencing (RNA-Seq) is the HTP NGS technique to sequence and quantify the abundance of mRNAs at whole genome level. RNA-Seq has been performed in various tissues and at different growth stages in maize under water stress condition. DEGs involved in cell wall biosynthesis, transmembrane, ROS scavenging, and ABA have been identified by subjecting the maize seedlings of the RIL population to drought stress (Min et al., 2016). By treating the leaf, stem and root of maize under well-watered and drought-stress conditions, 5,866 DEGs including eight MAPKKK genes responsive to drought stress have been identified (Liu et al., 2015). They also found that DEGs involved in oxidation, photosynthesis, starch, proline, ethylene, and salicylic acid metabolisms were co-expressed with the MAPKKK genes. Drought treatment to *Zea mays* ssp. *mexicana* L. (a member of teosinte, a wild relative of the *Zea mays* spp. *mays* L.) generated 614 DEGs through RNA-Seq analysis. Functional enrichment analyses showed that these DEGs were related to ABA, trehalose synthesis and ICE1-CBF pathways (Lu et al., 2017). Affymterix GenChip maize genome array, a hybridisation-based technique, comprising 17,555 probes has been used for identification of DEGs under drought stress (Zhang et al., 2010; Thirunavukkarasu et al., 2017). Customized oligo arrays having 1000 genes were used to identify the DEGs under drought stress in maize (Marino et al., 2009). HTP methods opened-up new possibilities to understand the expression of DEGs involved in various pathways under stress condition at genome-scale. The selected drought-responsive DEGs will be systematically validated in an independent set of genotypes, or in candidate-gene mapping experiments to further exploit them in breeding programmes. A set 52 drought–responsive candidate genes collected from the public data base and from the GWAS (Shikha et al., 2017) was validated in five maize hybrids and seven parental lines. Differential regulation and interactions of genes in various biological functions explained the basis of drought tolerance in subtropical maize hybrids (Van Gioi et al., 2017). A set of genes and their characteristics identified for drought tolerance through genomics and functional genomics approaches is presented in Table 1.

**Table 1:** List of drought stress-responsive genes and their characteristics controlling various important traits in maize.

**Trait**	**Gene ID**	**Gene name**	**Chr**	**Gene start**	**Gene end**	**Functional mechanism**	**References**
Stomatal regulation	GRMZM2G407181	*nced2*	1	174550907	174553815	ABA-dependent pathway	Iuchi et al., 2001; Thompson et al., 2007
	GRMZM2G089619	*zhd 15*	2	50140925	50142374	ABA-dependent pathway	Davletova et al., 2005
	GRMZM2G053384	*PRC protein*	2	227347436	227349815	RNA binding	Thirunavukkarasu et al., 2014; Van Gioi et al., 2017
	GRMZM2G102429	*u-box*	2	226617615	226619360	Catalytic activity	Thompson et al., 2007; Thirunavukkarasu et al., 2014
	GRMZM5G858784	*nced3*	3	87358369	87360132	ABA-dependent pathway	Iuchi et al., 2001; Thompson et al., 2007
	GRMZM2G159724	*me6*	3	201756751	201761835	Nucleotide binding, protein binding	Thirunavukkarasu et al., 2014; Van Gioi et al., 2017
	GRMZM2G069365	*zhd 17*	4	160153804	160155930	ABA-dependent pathway	Davletova et al., 2005
	GRMZM2G122479	*me2*	6	139464390	139470075	Ion homeostasis-dependent pathway	Laporte et al., 2002
	GRMZM2G161680	*ca5p3*	6	93659875	93665056	Stomatal conductance	Song et al., 2017
	GRMZM2G071112	*zhd 13*	7	112658777	112661470	ABA-dependent pathway	Davletova et al., 2005
Root developoment	GRMZM2G003466	*ereb101*	1	20094963	20096296	Dessication tolerance	Liu et al., 2013
	GRMZM2G124037	*dbf3*	2	194479918	194481002	Dessication tolerance	Liu et al., 2013
	GRMZM2G060465	*ereb155*	4	185036196	185037019	DNA binding	Thirunavukkarasu et al., 2014
	GRMZM2G090576	*nac3*	5	20815035	20817354	Auxin transport	Hund et al., 2009
	GRMZM2G432571	*NBS-IRR partial*	5	20914992	20918282	Nucleotide binding	Thirunavukkarasu et al., 2014; Van Gioi et al., 2017
	GRMZM2G028648	*nac2*	6	115935425	115937455	Auxin transport	Hund et al., 2009
	GRMZM2G104400	*nactf38*	8	102056994	102060989	Auxin transport	Hund et al., 2009
	GRMZM2G134073	*nac68*	8	160424732	160426914	DNA binding	Thirunavukkarasu et al., 2014; Van Gioi et al., 2017
	GRMZM2G015605	*nac1*	10	87283919	87284844	Auxin transport	Hund et al., 2009
	GRMZM2G091819	*Flavin monoxygenase*	10	16522572	16525775	Auxin biosynthesis	Overvoorde et al., 2010
	GRMZM2G371345	*V-type PPase H+ pump*	10	46790874	46793155	Auxin transport	Overvoorde et al., 2010
ROS	GRMZM2G066120	*mkkk11*	1	37470728	37476121	Reactive oxygen species homeostasis	Zhu, 2002
	GRMZM2G172322	*gsr1*	1	12985602	12991971	H_2_O_2_ metabolism	Galle et al., 2013
	GRMZM2G140667	*apx2*	2	219258176	219261097	Reactive oxygen species homeostasis	Badawi et al., 2004
	GRMZM2G054559	*pld1*	3	12195404	12200349	Phospholipid hydrolysis	Zhu, 2002
	GRMZM2G071021	*aldh3*	3	221771183	221775333	Reactive oxygen species homeostasis	Miao et al., 2006; Chen et al., 2012
	GRMZM2G367411	*mkk6*	5	12679871	12707265	Kinase activity, nucleotide binding	Thirunavukkarasu et al., 2014; Van Gioi et al., 2017
	GRMZM2G059991	*sod3*	6	136070517	136074741	Oxygen radical detoxification	McKersie et al., 1996; Castillejo et al., 2008
	GRMZM2G025992	*sod2*	7	171775019	171778224	Oxygen radical detoxification	McKersie et al., 1996; Castillejo et al., 2008
	GRMZM5G884600	*GPx*	10	138607002	138608876	Catalytic activity	Thirunavukkarasu et al., 2014; Van Gioi et al., 2017
	GRMZM5G822829	*BHLH*	10	138462252	138463015	DNA binding	Thirunavukkarasu et al., 2014; Van Gioi et al., 2017
Hormone Signaling	GRMZM2G083717	*wrky14*	1	299282977	299288556	Sequence-specific DNA binding	Thirunavukkarasu et al., 2014; Van Gioi et al., 2017
	GRMZM2G008250	*NFY-A*	1	174845979	174849344	Sequence-specific DNA binding	Thirunavukkarasu et al., 2014; Van Gioi et al., 2017
	GRMZM2G117851	*bzip1*	3	212179339	212194812	Sequence-specific DNA binding	Thirunavukkarasu et al., 2014; Van Gioi et al., 2017
	GRMZM2G056120	*artf11*	3	196638145	196644110	ABA-inducible TFs triggering stomatal closure	Furihata et al., 2006; Kim et al., 2012
	GRMZM2G172327	*myb14*	7	150087003	150088438	DNA binding,chromatin binding	Thirunavukkarasu et al., 2014; Van Gioi et al., 2017
	GRMZM2G152661	*camta5*	10	109572710	109580177	DNA binding, protein binding	Thirunavukkarasu et al., 2014; Van Gioi et al., 2017
Photosynthesis	GRMZM2G414192	*umc1383*	1	247786615	247788296	Chlorophyll A-B binding protein	Song et al., 2017
	GRMZM2G078409	*ploc2*	2	24166809	24167435	Electron transfer	Efeoglu et al., 2009
	GRMZM2G162200	*rca1*	4	693736	696087	Role in photosynthesis	Rollins et al., 2013
	GRMZM2G162282	*rca3*	4	691410	693139	Role in photosynthesis	Rollins et al., 2013
	GRMZM2G568636	*ferredoxin–nitrite reductase*	4	56335735	56340792	photosynthetic electron transport	Min et al., 2016
	GRMZM2G122337	*Ferredoxin 1*	6	1340417	1341388	Oxidation reduction process	Kimata and Hase, 1989
	GRMZM2G033885	*light harvesting complex photosystem II*	7	157314547	157315990	Photosynthesis antenna2	Min et al., 2016
	GRMZM2G012397	*psa6*	7	5134217	5135120	Photosystem I reaction center 6	Li et al., 2011
	GRMZM2G033208	*Transketolase*	9	22790749	22795294	Carbon fixation in photosynthetic organisms	Min et al., 2016
Sucrose metabolism	GRMZM2G175423	*sodh1*	1	197301249	197304338	Cellulose hydrolysis	Mei et al., 2009
	GRMZM2G391936	*ADP glucose pyrophosphorylase large subunit 1*	1	273514151	273518985	Starch synthesis	Min et al., 2016
	GRMZM2G028353	*cellulose synthase 6*	2	170393027	170398878	Cellulose synthase	Min et al., 2016
	GRMZM2G018241	*Cellulose synthase 9*	2	161757546	161763704	Cellulose synthase	Min et al., 2016
	GRMZM2G130043	*ss5*	4	172635729	172706662	Hydrolysis of sucrose	Ruan et al., 2010
	GRMZM2G122277	*cellulose-synthase like D2*	4	31271742	31278123	Cellulose synthase	Min et al., 2016
	GRMZM2G046587	*pyrophosphorylase 1*	5	200881599	200885907	starch synthesis	Min et al., 2016
	GRMZM2G089836	*invertase2*	5	67537393	67540691	Response to drought stress	Zhang et al., 2017
	GRMZM2G058310	*amyb5*	7	155396510	155399710	Starch degradation	Rizhsky et al., 2004
	GRMZM2G152908	*sus1*	9	122479052	122485725	Sucrose metabolism	Gonzalez et al., 1995
	GRMZM2G016890	*Sbe2A*	10	34240659	34246077	Starch biosynthesis	Hurkman et al., 2003
ABA-mediated ignaling	GRMZM2G057935	*phyC1*	1	277059620	277064623	Signaling network	Sheehan et al., 2004
	AC198979.4_FG009	*ereb95*	1	10988032	10988424	ABA-dependent signaling pathway	Zhang et al., 2017
	GRMZM2G082487	*ZmPP2C-A5*	2	176186305	176187719	ABA signaling	Xiang et al., 2017
	GRMZM2G019819	*ZmPP2C-A8*	2	103675901	103679774	ABA signaling	Xiang et al., 2017
	GRMZM2G059453	*ZmPP2C-A1*	3	180096139	180098099	ABA signaling	Xiang et al., 2017
	GRMZM5G867568	*MAPKK3*	3	111079872	111080021	ABA signaling	Zhang et al., 2012
	GRMZM2G122228	*ZmPP2C-A6*	3	212913668	212919667	ABA signaling	Xiang et al., 2017
	GRMZM2G134628	*ZmPP2C-A11*	3	220928320	220934085	ABA signaling	Xiang et al., 2017
	GRMZM2G112240	*prh1*	4	170944444	170947965	ABA signaling network	Zheng et al., 2010
	GRMZM2G137046	*bzip61*	5	112670143	112675317	ABA signaling	Song et al., 2017
	GRMZM2G066867	*snrkII10*	5	18469442	18472522	ABA signaling network	Schafleitner et al., 2007; Mao et al., 2010
	GRMZM2G177386	*ZmPP2C-A10*	6	161744096	161747484	ABA signaling	Xiang et al., 2017
	GRMZM2G102255	*ZmPP2C-A12*	6	167604993	167607983	ABA signaling	Xiang et al., 2017
	GRMZM2G308615	*ZmPP2C-A4*	7	89059718	89061311	ABA signaling	Xiang et al., 2017
	GRMZM2G069146	*ereb115*	7	141174421	141175245	ABA-dependent signaling pathway	Song et al., 2017
	GRMZM2G305066	*MKKK18*	8	152510200	152511639	Signaling network	Shou et al., 2004
	GRMZM2G166297	*ZmPP2C-A2*	8	168030486	168032471	ABA signaling	Xiang et al., 2017
	GRMZM5G818101	*ZmPP2C-A7*	8	72141439	72143820	ABA signaling	Xiang et al., 2017
	GRMZM2G383807	*ZmPP2CA-13*	8	77501631	77504702	ABA signaling	Xiang et al., 2017
	GRMZM2G180555	*MKKK10*	9	141628047	141638073	Signaling network	Shou et al., 2004
	GRMZM2G159811	*ZmPP2C-A9*	10	101377174	101382475	ABA signaling	Xiang et al., 2017
	GRMZM2G142718	*dof41*	10	147844469	147845926	ABA-dependent signaling pathway	Song et al., 2017
Aquaporins	GRMZM2G081843	*PIP1;4*	4	170004633	170006133	Aquarins	Min et al., 2016
	GRMZM2G154628	*PIP2.1*	5	195239679	195242694	Aquarins	Min et al., 2016
	GRMZM2G137108	*NOD26-like intrinsic protein 4*	6	113136258	113140405	Aquarins	Min et al., 2016
Delayed flowering time	GRMZM2G021777	*col3*	5	183418226	183419769	Delay flowering time	Song et al., 2017
	GRMZM2G004483	*cct2*	9	115786897	115789787	Delay flowering time	Song et al., 2017
Plant development	GRMZM2G336533	*nactf60*	5	2887335	2889124	DNA- binding	Zhang et al., 2017
	GRMZM2G024973	*d9*	5	11793473	11795945	Modulator of plant development	Zhang et al., 2017
	GRMZM2G126566	*myb159*	7	108815110	108818213	DNA binding,chromatin binding	Zhang et al., 2017
	GRMZM2G042666	*C2H2*	7	146276397	146281153	Plant development	Zhang et al., 2017
	GRMZM2G140355	*bzip80*	9	85130086	85132905	Sequence-specific DNA binding	Song et al., 2017
Signal transduction	GRMZM2G466563	–	1	220901789	220904352	Signal transduction	Alam et al., 2010
	GRMZM2G428554	–	1	210641732	210645615	Signal transduction	Perruc et al., 2004

Small RNAs called micro RNAs are the class of regulatory RNAs and their role in drought stress response through regulating the target mRNAs has been reported in maize. Genome-wide survey of miRNAs provided 150 high-confidence genes within 26 miRNA families (Zhang et al., 2009). A total of 192 mature miRNAs including 68 potential novel miRNA candidates was identified by constructing small RNA libraries at genome-scale from a set of contrasting maize genotypes to drought response. Five of these were differentially expressed under drought stress and played an important role in photosynthesis under drought stress (Sheng et al., 2015). A set of 13 drought-associated miRNA families regulating 42 unique target mRNAs were identified from drought-exposed seedlings of maize. The expression analysis revealed that miRNAs had both positive and negative regulations with their respective target mRNAs under stress (Aravind et al., 2017). RNA-Seq coupled with transcriptome re-assembly, a total of 13,387 long non-coding RNAs (lncRNAs) was identified under water-stress condition using maize seedlings. The identification of non-coding RNAs also revealed the role of epigenetic mechanism responsible for stress tolerance (Forestan et al., 2016).

Information on proteins and post translational modification under drought stress provide better knowledge on trait expression and selection of QTLs/genes for tolerance. Rapid quantification of proteins at genome-level is possible with the help of modern proteomics techniques such as shotgun proteomics [multidimensional protein identification technology (MudPIT)], isotope-code affinity tags (ICATs), targeted mass tags (TMTs), isobaric tags for relative and absolute quantitation (iTRAQ) (Ghatak et al., 2017). A set of 61 drought-associated proteins were identified at eight-leaf stage after exposing the maize plant to drought stress through iTRAQ approach. Functional characterization of these proteins revealed that chaperone proteins, proteases, ethylene responsive proteins and ripening-related proteins played a major role in drought tolerance (Zhao et al., 2016a). Using the same technique, 150 ABA-dependent proteins were identified from a set of ABA-deficient maize mutant Vp5 and its wild-type under drought stress (Zhao et al., 2016b). Using multiplex iTRAQ-based quantitative proteomic and LC-MS/MS methods, 149 differentially phosphorylated peptides were identified at five-leaf stage of maize under drought stress (Hu et al., 2015). Leaf proteome of maize under moderate drought were analyzed by two independent approaches, 2D gel electrophoresis and iTRAQ, revealed the importance of detoxification proteins in drought tolerance (Benešová et al., 2012).

The role of specific proteins have been identified and characterized under drought stress conditions in maize. Up-regulation of RAB 17, phosphoribulokinase, caffeate *O*-methyltransferase, COMT, glutamate semialdehyde aminotransferase (GSAAT), β-glucosidase, chloroplastic fructose bisphosphate aldolase, and ferritin proteins under drought stress condition were identified from the maize leaf tissue (Riccardi et al., 1998). Changes in the expression of oxygen evolving enhancer (OEE) protein 1, malate dehydrogenase and ABA stress ripening (ASR) proteins were identified from the leaf proteome under drought stress (Riccardi et al., 2004). Non accumulation of two isoforms (acidic protein COMT 1 and less acidic protein COMT 2) of caffeic acid/5-hydroxyferulic 3-Omethyltransferase in the drought-stressed maize leaves was identified as the cause for reduced leaf elongation under stress (Vincent et al., 2005).

Metabolites react with environmental changes and are the better candidates to study the drought response. Recent studies indicated that metabolites have a positive correlation with drought tolerance. Metabolic traits could be used as an additional selection tool along with other genomic tools to improve drought tolerance in maize. Gas chromatography-mass spectroscopy (GC-MS)-based metabolite profiling revealed 41 metabolites under drought stress of which glysine and myoinositol were significantly correlated with grain yield in maize (Obata et al., 2015). Tryptophan, proline, histidine, and several intermediates from the TCA cycle analyzed through GC–TOF–MS method showed significant difference in the maize hybrids under drought stress condition. These metabolites also had a strong relationship with phenotypic traits (Witt et al., 2012). A mass spectrometer analysis detected different levels of abscisic acid, jasmonate, salicylic acid, and other hormones in herbicide tolerant maize varieties under control and drought-stress conditions (Benevenuto et al., 2017).

The functional genomics approaches play important role to understand the identification of genes operating in stress tolerant pathways, interaction of key genes in various pathways and contribution of genes to final trait expression under stress condition. Together with genomics, functional genomics approaches are useful in selection of better genotypes in stress breeding programmes.

## Marker-based selection approaches

QTLs and genes are identified and validated by various trait mapping and functional studies. For the validated QTLs and genes, QTL-flanking and gene-specific markers can be designed. The markers are further used in applied breeding through MAS, MARS and GS to improve the trait expression.

### MAS

QTL-based selection techniques largely follow marker-assisted backcrossing (MABC) or MARS. Although several QTL mapping experiments are reported in maize little has been published on the successful introgression of QTLs especially for drought tolerance. A successful MABC programme by introgressing five QTLs for ASI in maize for drought tolerance has been reported (Ribaut and Ragot, 2007).

The success of MAS programmes for drought tolerance depends upon two components: 1. Identification of true QTLs for the component traits, 2. Introgression of the identified QTLs in MAS. Drought is a combination of several quantitative traits with a high level of epistatic and environmental interactions. Quantifying the phenotypic variation explained by the QTLs and their possible interactions are important issues since the effects are confounded with study design, selection of component traits, challenges involved in phenotyping, marker coverage, genotyping, QTL mapping models and so on. Any fluctuation on the above-mentioned factors would significantly alter the QTL numbers and their phenotypic contribution. Apart from this, though true QTLs are identified, practically, chasing too many QTLs through MABC remains a daunting task. So during the introgression programme, transferring one or two major QTLs do not provide the expected level of trait expression since a QTL identified with epistatic interaction lose the effect in the absence of its counterparts. All above-mentioned factors are to be considered while performing QTL mapping experiments since a successful MAS programme depends upon the QTL mapping results.

### MARS

MARS allows simultaneous identification and improvement of polygenic traits by stacking favorable alleles at a large number of the loci. MARS can be either used by inter-mating the marker genotypes in random (Hospital et al., 1997; Moreau et al., 1998) or directed recombination of the selected genotypes of a segregating population (Charmet et al., 1999). MARS was successfully employed in maize to improve the complex quantitative traits such as yield and stover quality (Massman et al., 2013) and drought tolerance (Beyene et al., 2015). Through MARS, the number of favorable alleles for the drought tolerance has been increased from 114 in C_0_ to 124 in C_3_ (Abdulmalik et al., 2017). The frequency of favorable alleles of drought tolerance was increased from 0.510 at C_0_ to 0.515 at C_2_ with a genetic gain of 3% by practicing MARS in maize (Bankole et al., 2017).

### GS

In the recent past, the genetics of traits was studied with the help of QTLs based on bi-parental mapping populations. This approach provides information on two alleles per locus. Later association mapping approach came up with a possibility of studying several alleles to the range of 30-40 depends upon the genetic variability present in the GWAS panel. Now, we have reached an era from studying a few loci to all loci of the genome (Figure 1). The GS models assume that all marker loci of the genome contribute to the trait-expression (Meuwissen et al., 2001) either positively or negatively, so small-effect marker loci will also be effectively included in the model (Heffner et al., 2009; Guo et al., 2012). This approach is quite useful to develop lines with best SNP combinations by combining SNPs from genetically diverse population. The cumulative effect of SNPs called as genomic estimated breeding value (GEBV) decides the expression of the trait. GS has two components: 1. Prediction of GEBVs and 2. Utilization of GEBVs in the selection programme. GEBVs can be predicted with the help of GS models using genome-wide SNPs and comprehensive phenotypic data. The best model was predicted using seven GS models in drought-phenotyped genotypes and compared the GS results with GWAS results (Shikha et al., 2017). Improvement of populations for drought tolerance through GS approach has been already reported in maize. About 7.3% higher grain yield in maize was obtained through GS over conventional selection under drought stress (Beyene et al., 2015). From 10 to 20% of GS was achieved over conventional phenotypic selection under drought conditions in the testcrosses from bi-parental populations using 1,214 SNP markers (Vivek et al., 2017).

**Figure 2:**
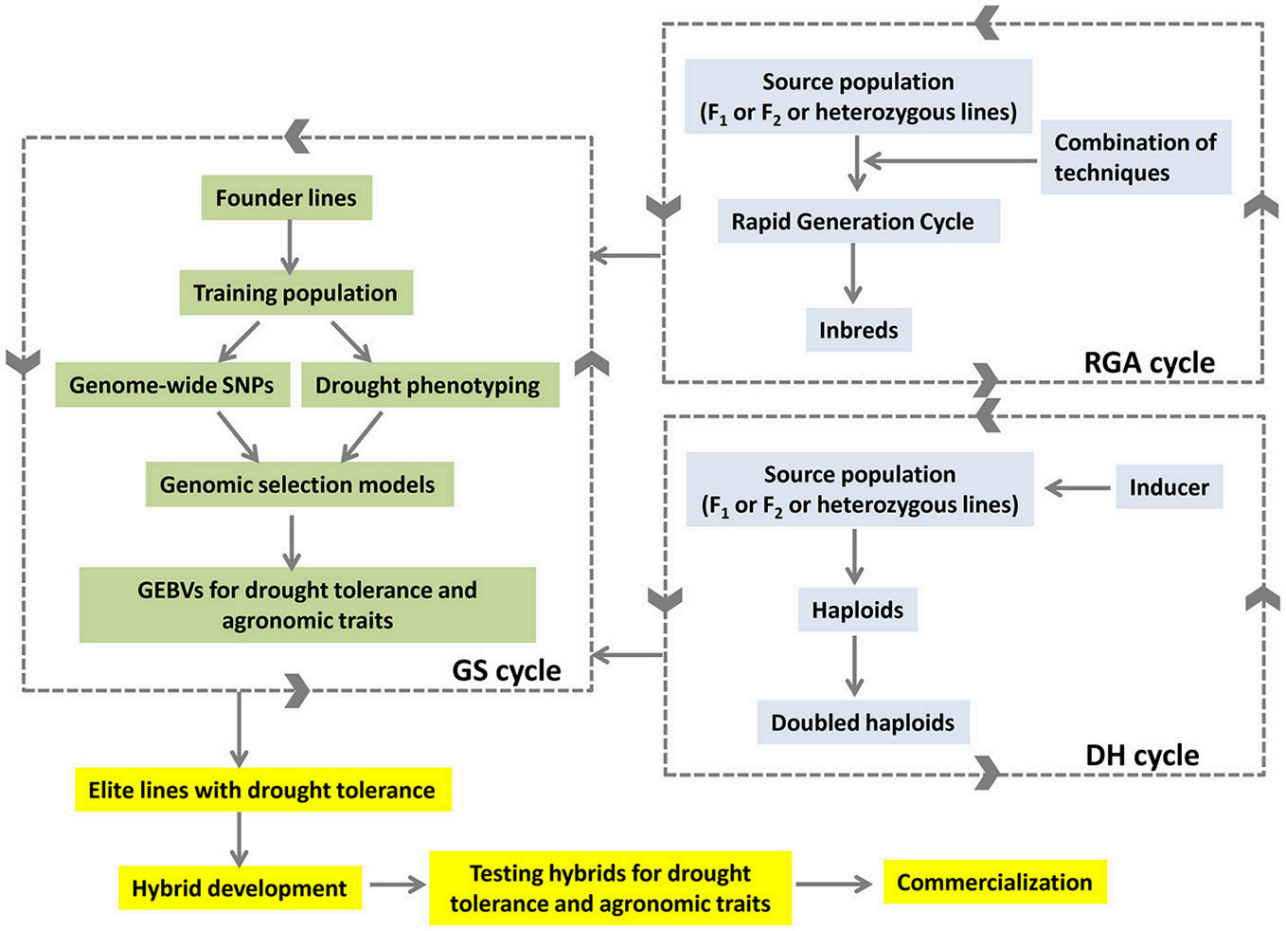
Accelerated development of drought tolerant hybrids by combining DH, RGA and GS approaches.

The combination of ALB with GS selection approaches is expected to reduce the breeding cycles and deliver the products rapidly in maize (Figure 2). The lines developed from ALB approach would feed back to the GS selection cycle to generate GEBVs. The GEBVs in turn would help in selection of better lines with drought tolerance developed through ALB. The selected elite lines would be used in hybrid breeding programme to develop drought-tolerant high-yielding hybrids.

## Genome editing

Targeted genome editing is the latest approach to manipulate the gene function. Of several approaches, clustered regularly interspaced short palindromic repeats (CRISPR) and CRISPR-associated protein9 nuclease (Cas9) are the effective genome editing technologies used in plant system (Gasiunas et al., 2012; Cong et al., 2013). Several modifications to the CRISPR/Cas9 system are now available for site-directed modifications such as truncated-gRNAs (tru-gRNAs) with no off-target effect (Osakabe et al., 2016). Zinc-finger nuclease (Kim et al., 1996) and transcription activator-like effector nucleases (TALENS) (Boch et al., 2009; Moscou and Bogdanove, 2009) are the other techniques used in editing the genes.

Through genome editing approaches, point mutation (deletion or insertion), gene knockouts, activation or repression of genes and epigenetic changes are possible (Kamburova et al., 2017). Many gene editing experiments are successful since the target traits are governed by a single gene. In maize upstream of the liguleless1 (LIG1) gene, male fertility genes (Ms26 and Ms45), and acetolactate synthase (ALS) genes (ALS1 and ALS2) have been successfully altered through targeted mutagenesis, precise gene editing, and site-specific gene insertion using Cas9 and guide RNA (Svitashev et al., 2015). A CRISPR/Cas9 binary vector set was developed as a toolkit to perform multiplex genome editing in a variety of plant species including maize (Xing et al., 2014). Biolistic delivery of pre-assembled Cas9–gRNA ribonucleoproteins into embryo cells, and DNA- and selectable marker-free recovery of plants with mutated alleles at high frequencies was demonstrated in maize (Svitashev et al., 2016). Recently, RNA editing using programmable single-effector RNA-guided RNases Cas13 has been reported (Cox et al., 2017). Through RNA editing the structure of the DNA remains intact whereas the function of genes is altered. The utility of RNA editing is yet to be explored in maize for drought tolerance.

Genome editing is currently akin to MAS where one or few genes are being modified for a given trait. Since drought tolerance is a complex trait, many genes need to be targeted to achieve the desired level of tolerance. Mutant lines can be created for various drought-responsive genes and through pyramiding approaches those genes can be introgressed into a single genotype. However, pyramiding approach is laborious and resource intensive. Hence, up-gradation or modification in the existing genome editing approaches is needed to alter several genes operating in various pathways in a genome at one go. Such an advanced approach will be useful to manipulate several target genes of individual and component traits to develop drought tolerant genotypes rapidly.

Combining genomics and gene editing techniques would accelerate the trait improvement at desired level. Through genomics one can understand the structure and function of the genes that are controlling simple and complex traits. The number, location, phenotypic contribution of genes to the trait expression should be elucidated in advance in order to use them in genome editing approaches. Since drought is controlled by several genes with variable level of phenotypic expression the epistatic interactions of genes should be thoroughly studied. This would help in selecting combination of genes to be targeted for editing experiment. Selection of genes that are working in tandem would be a good option for trait improvement program rather working on individual genes to realize improved genetic gain. Identification of candidate genes for drought through genomics followed by editing of those target genes is necessary to improve the genetic gain rapidly.

## Genome and breeding informatics, and big-data

The cost-effective ever-growing genomics approaches generated data in the form of DNA and RNA sequences, proteins, metabolites and etc. to the scale of 100's of terabytes. The large scale data produced from such genomic techniques is called as “Big data.” The large-scale genomics data will pose computational challenges thus new techniques need to be developed (Stephens et al., 2015). The management of big data includes storage, compilation, curation, processing, complex data analyses, visualization, retrieval and sharing. High-power local server- or cloud-based computing systems are necessary to manage the big data.

The genome-level big data developed from omics techniques are complementary to each other to understand the structural architecture of genome and functional complexities of gene regulation at intra and inter species level. Customised informatics platforms are needed to integrate the big data to get meaningful information and decisions. Genomics Open-source Breeding Informatics Initiative (GOBII) is one of such open-source platforms to develop and implement genomics data management tools (http://cbsugobii05.tc.cornell.edu/wordpress/).

The decisions from the big data will be exploited in the applied breeding programmes such as MAS, MARS, and GS. These selection programmes have several activities including phenotyping, genotyping, backcross breeding, testing the product and etc. Breeding informatics tools play handy to streamline the activities of the selection programmes and help in effective management of the breeding processes.

**Figure 3:**
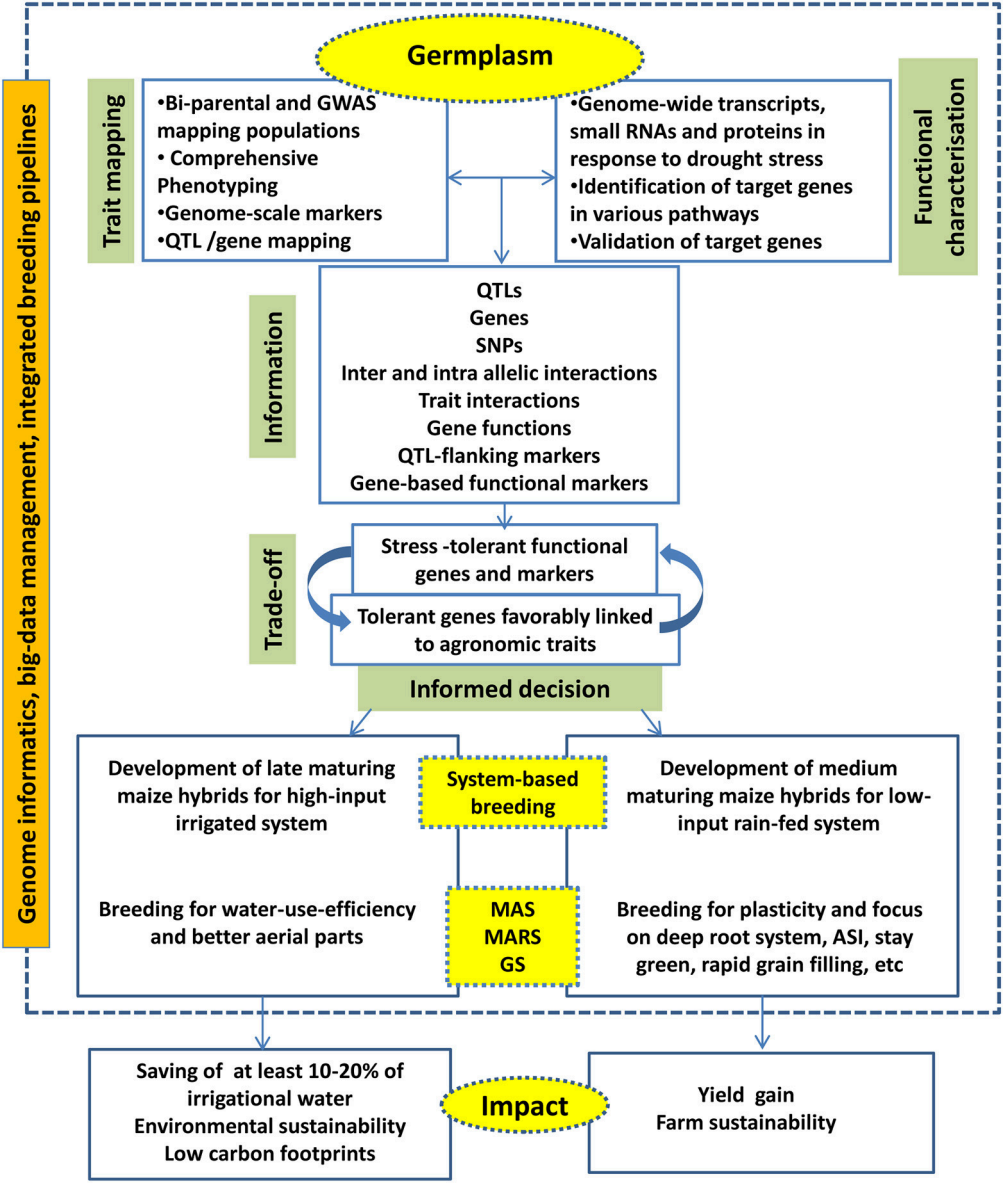
The next generation drought breeding involves utilization of various genome-level techniques, decision from different informatics pipelines to deliver products for system-specific hybrids.

Integration of big data management tools, decision making tools and down-stream molecular breeding activities are become necessary to practice the next-generation breeding efficiently and to accelerate the product delivery. Various commercial and open-source platforms are available to combine genomics, data management and breeding activities. Integrated Breeding Platform (www.integratedbreeding.net) has provided various informatics tools to manage genomics and breeding data. Complex trait breeding such as drought tolerance will be benefitted when various genomics, novel breeding, and informatics tools are combined effectively (Figure 3).

## Conclusions

Breeding for climate-resilient drought tolerant maize is important owing to changing climatic conditions. Though it is an important trait, but its complex inheritance poses a major challenge to the researchers. Several morpho-physiological traits have been reported for drought tolerance in maize. Since maize growing in different agro-climatic conditions, system-specific approach would be relevant to choose target traits for improvement. Genomics and breeding tools have come-up in the last two decades for better understanding of the inheritance of traits. Cost-effective third-generation sequencing technologies are now available to capture the SNPs as well as customize the genotyping. The identification and functional characterization of genes involved in various drought tolerance mechanisms can be performed with the help of expression and protein assays. Combining approaches such as DH technology with GS would be useful to accelerate the drought breeding in maize. The genomics and breeding approaches should be sufficiently complemented and supported with sophisticated informatics tools. Big data management and informatics tools have become necessary in applied breeding programmes. Genome editing approaches are going to play a big role in future in developing customized genotypes for the target environment. Identification of key genes operating in different pathways through QTL/gene mapping and gene expression assays remains important to exploit them in genome editing experiments. The editing of target genes could provide desired level of drought tolerance and sustain the grain yield in hybrids for different production systems. Although several advancements have happened in the field of genomics, knowledge on inter- and intra -allelic interactions need to be focussed to improve the heritability. In order to maximise the genetic gain in the selection programmes for drought tolerance, focus has to be given to elucidate the inter-trait interaction at molecular level. Precision breeding of drought tolerant maize hybrids is possible by strategic integration of modern genomics approaches with advanced breeding methods.

## Author contributions

TN conceived the concept; TN, JK, GM, and SM wrote the manuscript.

### Conflict of interest statement

The authors declare that the research was conducted in the absence of any commercial or financial relationships that could be construed as a potential conflict of interest.

## References

[B1] Abdulmalik R. O. Menkir A. Meseka S. K. Unachukwu N. Ado S. G. Olarewaju J. D.. (2017). Genetic gains in grain yield of a maize population improved through marker assisted recurrent selection under stress and non-stress conditions in West Africa. Front. Plant Sci. 8:841. 10.3389/fpls.2017.00841 28588598 PMC5438988

[B2] Agrama H. A. Moussa M. E. (1996). Mapping QTLs in breeding for drought tolerance in maize (*Zea mays L*.). Euphytica 91, 89–97. 10.1007/BF00035278

[B3] Alam M. M. Sharmin S. Nabi Z. Mondal S. I. Islam M. S. Nayeem S. B. (2010). A putative leucine-rich repeat receptor-like kinase of jute involved in stress response. Plant Mol. Biol. Report. 28, 394–402. 10.1007/s11105-009-0166-4

[B4] Almeida G. D. Makumbi D. Magorokosho C. Nair S. Borém A. Ribaut J. M.. (2013). QTL mapping in three tropical maize populations reveals a set of constitutive and adaptive genomic regions for drought tolerance. Theor. Appl. Genet. 126, 583–600. 10.1007/s00122-012-2003-7 23124431 PMC3579412

[B5] Araus J. L. Serret M. D. Edmeades G. O. (2012). Phenotyping maize for adaptation to drought. Front. Physiol. 3:305. 10.3389/fphys.2012.00305 22934056 PMC3429076

[B6] Aravind J. Rinku S. Pooja B. Shikha M. Kaliyugam S. Mallikarjuna M. G.. (2017). Identification, characterization, and functional validation of drought-responsive microRNAs in subtropical maize inbreds. Front. Plant Sci. 8:941. 10.3389/fpls.2017.00941 28626466 PMC5454542

[B7] Badawi G. H. Kawano N. Yamauchi Y. Shimada E. Sasaki R. Kubo A.. (2004). Over-expression of ascorbate peroxidase in tobacco chloroplasts enhances the tolerance to salt stress and water deficit. Physiol. Plant. 121, 231–238. 10.1111/j.0031-9317.2004.00308.x 15153190

[B8] Baird N. A. Etter P. D. Atwood T. S. Currey M. C. Shiver A. L. Lewis Z. A.. (2008). Rapid SNP discovery and genetic mapping using sequenced RAD markers. PLoS ONE 3:e3376. 10.1371/journal.pone.0003376 18852878 PMC2557064

[B9] Bankole F. Menkir A. Olaoye G. Crossa J. Hearne S. Unachukwu N.. (2017). Genetic gains in yield and yield related traits under drought stress and favorable environments in a maize population improved using marker assisted recurrent selection. Front. Plant Sci. 8:808. 10.3389/fpls.2017.00808 28567048 PMC5434104

[B10] Benešová M. Holá D. Fischer L. Jedelský P. L. Hnilička F. Wilhelmová N.. (2012). The physiology and proteomics of drought tolerance in Maize: early stomatal closure as a cause of lower tolerance to short-term dehydration? PLoS ONE 7:e38017. 10.1371/journal.pone.0038017 22719860 PMC3374823

[B11] Benevenuto R. F. Agapito-Tenfen S. Z. Vilperte V. Wikmark O. G. van Rensburg P. J. Nodari R. O. (2017). Molecular responses of genetically modified maize to abiotic stresses as determined through proteomic and metabolomic analyses. PLoS ONE 12:e0173069. 10.1371/journal.pone.0173069 28245233 PMC5330488

[B12] Beyene Y. Semagn K. Mugo S. Tarekegne A. Babu R. Meisel B. (2015). Genetic gains in grain yield through genomic selection in eight bi-parental maize populations under drought stress. Crop Sci. 55, 154–163. 10.2135/cropsci2014.07.0460

[B13] Boch J. Scholze H. Schornack S. Landgraf A. Hahn S. Kay S. (2009). Breaking the code of DNA binding specificity of TAL-Type III Effectors. Science 80, 1509–1512. 10.1126/science.1178811 19933107

[B14] Buckler E. S. Holland J. B. Bradbury P. J. Acharya C. B. Brown P. J. Browne C. (2009). The genetic architecture of maize flowering time. Science 80, 714–718. 10.1126/science.1174276 19661422

[B15] Bukowski R. Guo X. Lu Y. Zou C. He B. Rong Z.. (2015). Construction of the third generation *Zea mays* haplotype map. *bioRxiv* 26963. 10.1101/026963 29300887 PMC5890452

[B16] Cao S. Loladze A. Yuan Y. Wu Y. Zhang A. Chen J.. (2017). Genome-wide analysis of tar spot complex resistance in maize using genotyping-by-sequencing SNPs and whole-genome prediction. Plant Genome 10, 1–14. 10.3835/plantgenome2016.10.0099 28724072

[B17] Castillejo M. Á. Maldonado A. M. Ogueta S. Jorrín J. V. (2008). Proteomic analysis of responses to drought stress in sunflower (*Helianthus annuus*) leaves by 2DE gel electrophoresis and mass spectrometry. Open Proteomics J. 1, 59–71. 10.2174/1875039700801010059

[B18] Charmet G. Robert N. Perretant M. R. Gay G. Sourdille P. Groos C. (1999). Marker-assisted recurrent selection for cumulating additive and interactive QTLs in recombinant inbred lines. Theor. Appl. Genet. 99, 1143–1148. 10.1007/s001220051318

[B19] Chen L. Song Y. Li S. Zhang L. Zou C. Yu D. (2012). The role of WRKY transcription factors in plant abiotic stresses. Biochim. Biophys. Acta 1819, 120–128. 10.1016/j.bbagrm.2011.09.002 21964328

[B20] Chia J. M. Song C. Bradbury P. J. Costich D. de Leon N. Doebley J.. (2012). Maize HapMap2 identifies extant variation from a genome in flux. Nat. Genet. 44, 803–807. 10.1038/ng.2313 22660545

[B21] Coe E. H. (1959). A line of maize with high haploid frequency. Am. Nat. 93, 381–382. 10.1086/282098

[B22] Cong L. Ran F. A. Cox D. Lin S. Barretto R. Habib N.. (2013). Multiplex genome engineering using CRISPR/Cas systems. Science 339, 819–823. 10.1126/science.1231143 23287718 PMC3795411

[B23] Cook J. P. McMullen M. D. Holland J. B. Tian F. Bradbury P. Ross-Ibarra J.. (2012). Genetic architecture of maize kernel composition in the nested association mapping and inbred association panels. Plant Physiol. 158, 824–834. 10.1104/pp.111.185033 22135431 PMC3271770

[B24] Cox D. B. T. Gootenberg J. S. Abudayyeh O. O. Franklin B. Kellner M. J. Joung J. (2017). Science 358, 1019–1027. 10.1126/science.aaq0180 29070703 PMC5793859

[B25] Crossa J. Beyene Y. Kassa S. Pérez P. Hickey J. M. Chen C.. (2013). Genomic prediction in maize breeding populations with genotyping-by-sequencing. G3 3, 1903–1926. 10.1534/g3.113.008227 24022750 PMC3815055

[B26] Davletova S. Schlauch K. Coutu J. Mittler R. (2005). The zinc-finger protein Zat12 plays a central role in reactive oxygen and abiotic stress signaling in arabidopsis. Plant Physiol. 139, 847–856. 10.1104/pp.105.068254 16183833 PMC1256000

[B27] Dell'Acqua M. Gatti D. M. Pea G. Cattonaro F. Coppens F. Magris G.. (2015). Genetic properties of the MAGIC maize population: a new platform for high definition QTL mapping in *Zea mays*. Genome Biol. 16:167. 10.1186/s13059-015-0716-z 26357913 PMC4566846

[B28] De La Fuente G. N. Frei U. K. Lübberstedt T. (2013). Accelerating plant breeding. Trends Plant Sci. 18, 667–672. 10.1016/j.tplants.2013.09.001 24080381

[B29] Edmeades G. (2008). Drought tolerance in maize: an emerging reality. A Feature In James, Clive. 2008. Global Status of Commercialized Biotech/GM Crops: 2008. Glob. Status Commer. Biotech/GM Crop. ISAAA Br. No. 39. Available online at: http://www.salmone.org/wp-content/uploads/2009/02/droughtmaize.pdf

[B30] Edmeades G. O. Bolaños J. Chapman S. C. Lafitte H. R. Bänziger M. (1999). Selection improves drought tolerance in tropical maize populations: I. gains in biomass, grain yield, harvest index. Crop Sci. 39, 1306–1315. 10.2135/cropsci1999.3951306x

[B31] Efeoglu B. Ekmekci Y. Cicek N. (2009). Physiological responses of three maize cultivars to drought stress and recovery. South Afr. J. Bot. 75, 34–42. 10.1016/j.sajb.2008.06.005

[B32] Elshire R. J. Glaubitz J. C. Sun Q. Poland J. A. Kawamoto K. Buckler E. S.. (2011). A robust, simple genotyping-by-sequencing (GBS) approach for high diversity species. PLoS ONE 6:e19379. 10.1371/journal.pone.0019379 21573248 PMC3087801

[B33] Forestan C. Aiese Cigliano R. Farinati S. Lunardon A. Sanseverino W. Varotto S.. (2016). Stress-induced and epigenetic-mediated maize transcriptome regulation study by means of transcriptome reannotation and differential expression analysis. Sci. Rep. 6:30446. 10.1038/srep30446 27461139 PMC4962059

[B34] Frova C. Krajewski P. di Fonzo N. Villa M. Sari-Gorla M. (1999). Genetic analysis of drought tolerance in maize by molecular markers I. Yield components. Theor. Appl. Genet. 99, 280–288. 10.1007/s001220051233

[B35] Furihata T. Maruyama K. Fujita Y. Umezawa T. Yoshida R. Shinozaki K.. (2006). Abscisic acid-dependent multisite phosphorylation regulates the activity of a transcription activator AREB1. Proc. Natl. Acad. Sci. U.S.A. 103, 1988–1993. 10.1073/pnas.0505667103 16446457 PMC1413621

[B36] Gallé Á. Csiszar J. Benyo D. Laskay G. Leviczky T. Erdei L.. (2013). Isohydric and anisohydric strategies of wheat genotypes under osmotic stress: biosynthesis and function of ABA in stress responses. J. Plant Physiol. 170, 1389–1399. 10.1016/j.jplph.2013.04.010 23702247

[B37] Gasiunas G. Barrangou R. Horvath P. Siksnys V. (2012). Cas9-crRNA ribonucleoprotein complex mediates specific DNA cleavage for adaptive immunity in bacteria. Proc. Natl. Acad. Sci. U.S.A. 109, E2579–E2586. 10.1073/pnas.1208507109 22949671 PMC3465414

[B38] Gaur P. M. Srinivasan S. Gowda C. L. L. Rao B. V (2007). Rapid generation advancement in chickpea. J. SAT Agric. Res. 3, 1–3.

[B39] Geiger H. H. Gordillo G. A. (2009). Double haploids in hybrid maize breeding. Maydica 54, 485–499.

[B40] Ghatak A. Chaturvedi P. Weckwerth W. (2017). Cereal crop proteomics: systemic analysis of crop drought stress responses towards marker-assisted selection breeding. Front. Plant Sci. 8:757. 10.3389/fpls.2017.00757 28626463 PMC5454074

[B41] Gonzalez E. M. Gordon A. J. James C. L. Arrese-lgor C. (1995). The role of sucrose synthase in the response of soybean nodules to drought. J. Exp. Bot. 46, 1515–1523. 10.1093/jxb/46.10.1515

[B42] Gore M. A. Chia J.-M. Elshire R. J. Sun Q. Ersoz E. S. Hurwitz B. L. (2009). A first-generation haplotype map of maize. Science 80, 1115–1117. 10.1126/science.1177837 19965431

[B43] Grift T. E. Novais J. Bohn M. (2011). High-throughput phenotyping technology for maize roots. Biosyst. Eng. 110, 40–48. 10.1016/j.biosystemseng.2011.06.004

[B44] Guo J. Su G. Zhang J. Wang G. (2008). Genetic analysis and QTL mapping of maize yield and associate agronomic traits under semi-arid land condition. Afr. J. Biotechnol. 7, 1829–1838. 10.5897/AJB2008.000-5031

[B45] Guo Z. Tucker D. M. Lu J. Kishore V. Gay G. (2012). Evaluation of genome-wide selection efficiency in maize nested association mapping populations. Theor. Appl. Genet. 124, 261–275. 10.1007/s00122-011-1702-9 21938474

[B46] Heffner E. L. Sorrells M. E. Jannink J. (2009). Genomic selection for crop improvement. Crop Sci. 49, 1–12. 10.2135/cropsci2008.08.0512

[B47] Hirsch C. N. Hirsch C. D. Brohammer A. B. Bowman M. J. Soifer I. Barad O.. (2016). Draft assembly of elite inbred line PH207 provides insights into genomic and transcriptome diversity in maize. Plant Cell 28, 2700–2714. 10.1105/tpc.16.00353 27803309 PMC5155341

[B48] Hoisington D. Khairallah M. Reeves T. Ribaut J. M. Skovmand B. Taba S.. (1999). Plant genetic resources: what can they contribute toward increased crop productivity? Proc. Natl. Acad. Sci. U.S.A. 96, 5937–5943. 10.1073/pnas.96.11.5937 10339521 PMC34209

[B49] Hospital F. Moreau L. Lacoudre F. Charcosset A. Gallais A. (1997). More on the efficiency of marker-assisted selection. Theor. Appl. Genet. 95, 1181–1189. 10.1007/s001220050679

[B50] Hu X. Wu L. Zhao F. Zhang D. Li N. Zhu G.. (2015). Phosphoproteomic analysis of the response of maize leaves to drought, heat and their combination stress. Front. Plant Sci. 6:298. 10.3389/fpls.2015.00298 25999967 PMC4419667

[B51] Hund A. Trachsel S. Stamp P. (2009). Growth of axile and lateral roots of maize: I development of a phenotying platform. Plant Soil 325, 335–349. 10.1007/s11104-009-9984-2

[B52] Hurkman W. J. McCue K. F. Altenbach S. B. Korn A. Tanaka C. K. Kothari K. M. (2003). Effect of temperature on expression of genes encoding enzymes for starch biosynthesis in developing wheat endosperm. Plant Sci. 164, 873–881. 10.1016/S0168-9452(03)00076-1

[B53] Iuchi S. Kobayashi M. Taji T. Naramoto M. Seki M. Kato T.. (2001). Regulation of drought tolerance by gene manipulation of 9-cis-epoxycarotenoid dioxygenase, a key enzyme in abscisic acid biosynthesis in Arabidopsis. Plant J. 27, 325–333. 10.1046/j.1365-313x.2001.01096.x 11532178

[B54] Jiao W. B. Schneeberger K. (2017). The impact of third generation genomic technologies on plant genome assembly. Curr. Opin. Plant Biol. 36, 64–70. 10.1016/j.pbi.2017.02.002 28231512

[B55] Kamburova V. S. Nikitina E. V. Shermatov S. E. Buriev Z. T. Kumpatla S. P. Emani C. (2017). Genome editing in plants: an overview of tools and applications. Int. J. Agron. 2017:15 10.1155/2017/7315351

[B56] Kim M. J. Park M.-J. Seo P. J. Song J.-S. Kim H.-J. Park C.-M. (2012). Controlled nuclear import of the transcription factor NTL6 reveals a cytoplasmic role of SnRK2.8 in the drought-stress response. Biochem. J. 448, 353–363. 10.1042/BJ20120244 22967043

[B57] Kim Y. G. Cha J. Chandrasegaran S. (1996). Hybrid restriction enzymes: zinc finger fusions to Fok I cleavage domain. Proc. Natl. Acad. Sci. U.S.A. 93, 1156–1160. 10.1073/pnas.93.3.1156 8577732 PMC40048

[B58] Kimata Y. Hase T. (1989). Localization of ferredoxin isoproteins in mesophyll and bundle sheath cells in maize leaf. Plant Physiol. 89, 1193–1197. 10.1104/pp.89.4.1193 16666683 PMC1055995

[B59] Klose R. Penlington J. Ruckelshausen A. (2009). Usability of 3D time-of-flight cameras for automatic plant phenotyping. Bornimer Agrartech. Berichte 69, 93–105.

[B60] Kover P. X. Valdar W. Trakalo J. Scarcelli N. Ehrenreich I. M. Purugganan M. D.. (2009). A multiparent advanced generation inter-cross to fine-map quantitative traits in *Arabidopsis thaliana*. PLoS Genet. 5:e1000551. 10.1371/journal.pgen.1000551 19593375 PMC2700969

[B61] Kump K. L. Bradbury P. J. Wisser R. J. Buckler E. S. Belcher A. R. Oropeza-Rosas M. A.. (2011). Genome-wide association study of quantitative resistance to southern leaf blight in the maize nested association mapping population. Nat. Genet. 43, 163–168. 10.1038/ng.747 21217757

[B62] Laporte M. M. Shen B. Tarczynski M. C. (2002). Engineering for drought avoidance: expression of maize NADP malic enzyme in tobacco results in altered stomatal function. J. Exp. Bot. 53, 699–705. 10.1093/jexbot/53.369.699 11886890

[B63] Lebreton C. Lazic-Jancic V. Steed A. Pekic S. Quarrie S. A. (1995). Identification of QTL for drought responses in maize and their use in testing causal relationships between traits. J. Exp. Bot. 46:853 10.1093/jxb/46.7.853

[B64] Lee E. A. Tollenaar M. (2007). Physiological basis of successful breeding strategies for maize grain yield. Crop Sci. 47(Suppl. 3):S-202–S-215. 10.2135/cropsci2007.04.0010IPBS

[B65] Li C. Li Y. Sun B. Peng B. Liu C. Liu Z. (2013). Quantitative trait loci mapping for yield components and kernel-related traits in multiple connected RIL populations in maize. Euphytica 193, 303–316. 10.1007/s10681-013-0901-7

[B66] Li J. Zhang Y. Gu J. Guo C. Wen S. Liu G. (2011). Molecular characterization and roles of AP2 transcription factors on drought tolerance in plants. Front. Agric. China 5, 463–472. 10.1007/s11703-011-1148-5

[B67] Li X. Zhou Z. Ding J. Wu Y. Zhou B. Wang R.. (2016). Combined linkage and association mapping reveals qtl and candidate genes for plant and ear height in maize. Front. Plant Sci. 7:833. 10.3389/fpls.2016.00833 27379126 PMC4908132

[B68] Liebisch F. Kirchgessner N. Schneider D. Walter A. Hund A. (2015). Remote, aerial phenotyping of maize traits with a mobile multi-sensor approach. Plant Methods 11, 9. 10.1186/s13007-015-0048-8 25793008 PMC4365514

[B69] Liu S. Wang X. Wang H. Xin H. Yang X. Yan J.. (2013). Genome-wide analysis of ZmDREB genes and their association with natural variation in drought tolerance at seedling stage of *Zea mays* L. PLoS Genet. 9:e1003790. 10.1371/journal.pgen.1003790 24086146 PMC3784558

[B70] Liu Y. Zhou M. Gao Z. Ren W. Yang F. He H.. (2015). RNA-seq analysis reveals MAPKKK family members related to drought tolerance in maize. PLoS ONE 10:0143128. 10.1371/journal.pone.0143128 26599013 PMC4658043

[B71] Lorenz A. Hoegemeyer T. (2013). The phylogenetic relationships of US maize germplasm. Nat. Genet. 45, 844–845. 10.1038/ng.2697 23892661

[B72] Lu G. Gao C. Zheng X. Han B. (2009). Identification of OsbZIP72 as a positive regulator of ABA response and drought tolerance in rice. Planta 229, 605–615. 10.1007/s00425-008-0857-3 19048288

[B73] Lu X. Zhou X. Cao Y. Zhou M. McNeil D. Liang S.. (2017). RNA-seq analysis of cold and drought responsive transcriptomes of *Zea mays* ssp. mexicana L. Front. Plant Sci. 8:136. 10.3389/fpls.2017.00136 28223998 PMC5293773

[B74] Maazou A. S. Jialu T. Qiu J. Liu Z. (2016). Breeding for drought tolerance in maize (*Zea mays* L.). Am. J. Plant Sci. 7:13 10.4236/ajps.2016.714172

[B75] Mao X. Zhang H. Tian S. Chang X. Jing R. (2010). TaSnRK2.4, an SNF1-type serine/threonine protein kinase of wheat (*Triticum aestivum* L.), confers enhanced multistress tolerance in Arabidopsis. J. Exp. Bot. 61, 683–696. 10.1093/jxb/erp331 20022921 PMC2814103

[B76] Marino R. Ponnaiah M. Krajewski P. Frova C. Gianfranceschi L. Pè M. E.. (2009). Addressing drought tolerance in maize by transcriptional profiling and mapping. Mol. Genet. Genomics 281, 163–179. 10.1007/s00438-008-0401-y 19018570

[B77] Massman J. M. Jung H. J. G. Bernardo R. (2013). Genomewide selection versus marker-assisted recurrent selection to improve grain yield and stover-quality traits for cellulosic ethanol in maize. Crop Sci. 53, 58–66. 10.2135/cropsci2012.02.0112

[B78] McKersie B. D. Bowley S. R. Harjanto E. Leprince O. (1996). Water-deficit tolerance and field performance of transgenic alfalfa overexpressing superoxide dismutase. Plant Physiol. 111, 1177–1181. 10.1104/pp.111.4.1177 12226355 PMC160994

[B79] Mei C. Park S. H. Sabzikar R. Qi C. Ransom C. Sticklen M. (2009). Green tissue-specific production of a microbial endo-cellulase in maize (*Zea mays* L.) endoplasmic-reticulum and mitochondria converts cellulose into fermentable sugars. J. Chem. Technol. Biotechnol. 84, 689–695. 10.1002/jctb.2100

[B80] Messmer R. Fracheboud Y. Bänziger M. Vargas M. Stamp P. Ribaut J. M. (2009). Drought stress and tropical maize: QTL-by-environment interactions and stability of QTLs across environments for yield components and secondary traits. Theor. Appl. Genet. 119, 913–930. 10.1007/s00122-009-1099-x 19597726

[B81] Meuwissen T. H. Hayes B. J. Goddard M. E. (2001). Prediction of total genetic value using genome-wide dense marker maps. Genetics 157, 1819–1829. 11290733 10.1093/genetics/157.4.1819 PMC1461589

[B82] Mhike X. Okori P. Magorokosho C. Ndlela T. (2012). Validation of the use of secondary traits and selection indices for drought tolerance in tropical maize (*Zea mays* L.). Afr. J. Plant Sci. 6, 96–102. 10.5897/AJPS11.179

[B83] Miao Y. Lv D. Wang P. Wang X.-C. Chen J. Miao C.. (2006). An Arabidopsis glutathione peroxidase functions as both a redox transducer and a scavenger in abscisic acid and drought stress responses. Plant Cell 18, 2749–2766. 10.1105/tpc.106.044230 16998070 PMC1626619

[B84] Min H. Chen C. Wei S. Shang X. Sun M. Xia R.. (2016). Identification of drought tolerant mechanisms in maize seedlings based on transcriptome analysis of recombination inbred lines. Front. Plant Sci. 7:1080. 10.3389/fpls.2016.01080 27507977 PMC4961006

[B85] Monneveux P. Sanchez C. Tiessen A. (2008). Future progress in drought tolerance in maize needs new secondary traits and cross combinations. J. Agric. Sci. 146, 287–300. 10.1017/S0021859608007818

[B86] Monneveux P. Sánchez C. Beck D. Edmeades G. O. (2006). Drought tolerance improvement in tropical maize source populations: evidence of progress. Crop Sci. 46, 180–191. 10.2135/cropsci2005.04-0034

[B87] Moreau L. Charcosset A. Hospital F. Gallais A. (1998). Marker-assisted selection effciency in populations of finite size. Genetics 148, 1353–1365. 9539448 10.1093/genetics/148.3.1353 PMC1460046

[B88] Moscou M. J. Bogdanove A. J. (2009). A simple cipher governs dna recognition by TAL effectors. Science 80, 1501–1501. 10.1126/science.1178817 19933106

[B89] Muraya M. M. Schmutzer T. Ulpinnis C. Scholz U. Altmann T. (2015). Targeted sequencing reveals large-scale sequence polymorphism in maize candidate genes for biomass production and composition. PLoS ONE 10:e0132120. 10.1371/journal.pone.0132120 26151830 PMC4495061

[B90] Nagel K. A. Putz A. Gilmer F. Heinz K. Fischbach A. Pfeifer J. (2012). GROWSCREEN-Rhizo is a novel phenotyping robot enabling simultaneous measurements of root and shoot growth for plants grown in soil-filled rhizotrons. Funct. Plant Biol. 39, 891–904. 10.1071/FP12023 32480839

[B90a] Nepolean T. Singh I. Hossain F. Pandey N. Gupta H. S. (2013). Molecular characterization and assessment of genetic diversity of inbred lines showing variability for drought tolerance in maize. J. Plant Biochem. Biotechnol. 22, 71–79 10.1007/s13562-012-0112-7

[B91] Obata T. Witt S. Lisec J. Palacios-Rojas N. Florez-Sarasa I. Yousfi S. Luis Araus J.. (2015). Metabolite profiles of maize leaves in drought, heat, and combined stress field trials reveal the relationship between metabolism and grain yield. Plant Physiol. 169, 2665–2683. 10.1104/pp.15.01164 26424159 PMC4677906

[B92] Osakabe Y. Watanabe T. Sugano S. S. Ueta R. Ishihara R. Shinozaki K.. (2016). Optimization of CRISPR/Cas9 genome editing to modify abiotic stress responses in plants. Sci. Rep. 6:26685. 10.1038/srep26685 27226176 PMC4880914

[B93] Overvoorde P. Fukaki H. Beeckman T. (2010). Auxin control of root development. Cold Spring Harb. Perspect. Biol. 2:a001537. 10.1101/cshperspect.a001537 20516130 PMC2869515

[B94] Perruc E. Charpenteau M. Ramirez B. C. Jauneau A. Galaud J.-P. Ranjeva R.. (2004). A novel calmodulin-binding protein functions as a negative regulator of osmotic stress tolerance in *Arabidopsis thaliana* seedlings. Plant J. 38, 410–420. 10.1111/j.1365-313X.2004.02062.x 15086802

[B95] Poland J. A. Bradbury P. J. Buckler E. S. Nelson R. J. (2011). Genome-wide nested association mapping of quantitative resistance to northern leaf blight in maize. Proc. Natl. Acad. Sci. U.S.A. 108, 6893–6898. 10.1073/pnas.1010894108 21482771 PMC3084105

[B96] Prigge V. Schipprack W. Mahuku G. Atlin G. N. Melchinger A. E. (2012). Development of *in vivo* haploid inducers for tropical maize breeding programs. Euphytica 185, 481–490. 10.1007/s10681-012-0657-5

[B97] Ribaut J. M. Ragot M. (2007). Marker-assisted selection to improve drought adaptation in maize: the backcross approach, perspectives, limitations, and alternatives. J. Exp. Bot. 58, 351–360. 10.1093/jxb/erl214 17158111

[B98] Ribaut J.-M. Jiang C. Gonzalez-de-Leon D. Edmeades G. O. Hoisington D. (1997). Identification of quantitative trait loci under drought conditions in tropical maize. 2. Yield components and marker-assisted selection strategies. Theor. Appl. Genet. 94, 887–896. 10.1007/s001220050492

[B99] Riccardi F. Gazeau P. de Vienne D. Zivy M. (1998). Protein changes in response to progressive water deficit in maize. Plant Physiol. 117, 1253–1263. 10.1104/pp.117.4.1253 9701581 PMC34889

[B100] Riccardi F. Gazeau P. Jacquemot M. P. Vincent D. Zivy M. (2004). Deciphering genetic variations of proteome responses to water deficit in maize leaves. Plant Physiol. Biochem. 42, 1003–1011. 10.1016/j.plaphy.2004.09.009 15707837

[B101] Rizal G. Karki S. Alcasid M. Montecillo F. Acebron K. Larazo N. (2014). Shortening the breeding cycle of sorghum, a model crop for research. Crop Sci. 54, 520–529. 10.2135/cropsci2013.07.0471

[B102] Rizhsky L. Davletova S. Liang H. Mittler R. (2004). The zinc finger protein Zat12 is required for cytosolic ascorbate peroxidase 1 expression during oxidative stress in Arabidopsis. J. Biol. Chem. 279, 11736–11743. 10.1074/jbc.M313350200 14722088

[B103] Rober F. K. Gordillo G. A. Geiger H. H. (2005). *In vivo* haploid induction in maize - performance of new inducers and significance of doubled haploid lines in hybrid breeding. Maydica 50, 275–283.

[B104] Rollins J. A. Habte E. Templer S. E. Colby T. Schmidt J. Von Korff M. (2013). Leaf proteome alterations in the context of physiological and morphological responses to drought and heat stress in barley (*Hordeum vulgare* L.). J. Exp. Bot. 64, 3201–3212. 10.1093/jxb/ert158 23918963 PMC3733145

[B105] Ruan Y. L. Jin Y. Yang Y. J. Li G. J. Boyer J. S. (2010). Sugar input, metabolism, and signaling mediated by invertase: roles in development, yield potential, and response to drought and heat. Mol. Plant 3, 942–955. 10.1093/mp/ssq044 20729475

[B106] Schafleitner R. Gutierrez Rosales R. O. Gaudin A. Alvarado Aliaga C. A. Martinez G. N. Tincopa Marca L. R.. (2007). Capturing candidate drought tolerance traits in two native Andean potato clones by transcription profiling of field grown plants under water stress. Plant Physiol. Biochem. 45, 673–690. 10.1016/j.plaphy.2007.06.003 17764965

[B107] Seckler D. Amarasinghe U. Molden D. De Silva R. Barker R. (1998). World Water Demand and Supply, 1990 to 2025: Scenarios and Issues. Colombo: International Water Management Institute, 1–40.

[B108] Semagn K. Beyene Y. Warburton M. L. Tarekegne A. Mugo S. Meisel B.. (2013). Meta-analyses of QTL for grain yield and anthesis silking interval in 18 maize populations evaluated under water-stressed and well-watered environments. BMC Genomics 14:313. 10.1186/1471-2164-14-313 23663209 PMC3751468

[B109] Sheehan M. J. Farmer P. R. Brutnell T. P. (2004). Structure and expression of maize phytochrome family homeologs. Genetics 167, 1395–1405. 10.1534/genetics.103.026096 15280251 PMC1470959

[B110] Sheng L. Chai W. Gong X. Zhou L. Cai R. Li X.. (2015). Identification and characterization of novel maize mirnas involved in different genetic background. Int. J. Biol. Sci. 11, 781–793. 10.7150/ijbs.11619 26078720 PMC4466459

[B111] Shikha M. Kanika A. Rao A. R. Mallikarjuna M. G. Gupta H. S. Nepolean T. (2017). Genomic selection for drought tolerance using genome-wide SNPs in maize. Front. Plant Sci. 8:550. 10.3389/fpls.2017.00550 28484471 PMC5399777

[B112] Shou H. Bordallo P. Wang K. (2004). Expression of the Nicotiana protein kinase (NPK1) enhanced drought tolerance in transgenic maize. J. Exp. Bot. 55, 1013–1019. 10.1093/jxb/erh129 15073214

[B113] Song K. Kim H. C. Shin S. Kim K.-H. Moon J.-C. Kim J. Y.. (2017). Transcriptome analysis of flowering time genes under drought stress in maize leaves. Front. Plant Sci. 8:267. 10.3389/fpls.2017.00267 28298916 PMC5331056

[B114] Spielbauer G. Armstrong P. Baier J. W. Allen W. B. Richardson K. Shen B. (2009). High-throughput near-infrared reflectance spectroscopy for predicting quantitative and qualitative composition phenotypes of individual maize kernels. Cereal Chem. 86, 556–564. 10.1094/CCHEM-86-5-0556

[B115] Stephens Z. D. Lee S. Y. Faghri F. Campbell R. H. Zhai C. Efron M. J. (2015). Big data: astronomical or genomical? PLoS Biol. 13:e1002195 10.1371/journal.pbio.1002195 26151137 PMC4494865

[B116] Svitashev S. Schwartz C. Lenderts B. Young J. K. Mark Cigan A. (2016). Genome editing in maize directed by CRISPR–Cas9 ribonucleoprotein complexes. Nat. Commun. 7:13274. 10.1038/ncomms13274 27848933 PMC5116081

[B117] Svitashev S. Young J. K. Schwartz C. Gao H. Falco S. C. Cigan A. M. (2015). Targeted mutagenesis, precise gene editing, and site-specific gene insertion in maize using Cas9 and guide RNA. Plant Physiol. 169, 931–945. 10.1104/pp.15.00793 26269544 PMC4587463

[B118] Tamaki H. Mitsuhashi S. Kudoh H. Nagano A. J. Yasugi M. (2016). Genomewide molecular polymorphisms among maize (*Zea mays* L.) inbred lines found from restriction-associated dna tag sequencing (RAD-Seq) analysis as a preliminary study on “genomewide selection” for breeding by japanese public sectors. Bull. NARO Inst. Livest. Grassl. Sci. 16, 1–9.

[B119] Tanaka J. Hayashi T. Iwata H. (2016). A practical, rapid generation-advancement system for rice breeding using simplified biotron breeding system. Breed. Sci. 66, 542–551. 10.1270/jsbbs.15038 27795679 PMC5010295

[B120] Thirunavukkarasu N. Hossain F. Kaliyugam S. Swati M. Kanika A. Abhishek A.. (2013). Unraveling the genetic architecture of subtropical maize (*Zea mays* L.) lines and their utility in breeding programs. BMC Genomics 14:877. 10.1186/1471-2164-14-877 24330649 PMC3867671

[B121] Thirunavukkarasu N. Hossain F. Arora K. Sharma R. Shiriga K. Mittal S.. (2014). Functional mechanisms of drought tolerance in subtropical maize (*Zea mays* L.) identified using genome-wide association mapping. BMC Genomics 15:1182. 10.1186/1471-2164-15-1182 25539911 PMC4367829

[B122] Thirunavukkarasu N. Sharma R. Singh N. Shiriga K. Mohan S. Mittal S.. (2017). Genomewide expression and functional interactions of genes under drought stress in maize. Int. J. Genomics 2017, 1–14. 10.1155/2017/2568706 28326315 PMC5343257

[B124] Thompson A. J. Mulholland B. J. Jackson A. C. McKee J. M. T. Hilton H. W. Symonds R. C.. (2007). Regulation and manipulation of ABA biosynthesis in roots. Plant Cell Environ. 30, 67–78. 10.1111/j.1365-3040.2006.01606.x 17177877

[B125] Thompson A. M. Crants J. Schnable P. S. Yu J. Timmermans M. C. P. Springer N. M.. (2014). Genetic control of maize shoot apical meristem architecture. G3 4, 1327–1337. 10.1534/g3.114.011940 24855316 PMC4455781

[B126] Tian H.-L. Wang F.-G. Zhao J.-R. Yi H.-M. Wang L. Wang R.. (2015). Development of maizeSNP3072, a high-throughput compatible SNP array, for DNA fingerprinting identification of Chinese maize varieties. Mol. Breed. 35:136. 10.1007/s11032-015-0335-0 26052247 PMC4449932

[B127] Tuberosa R. Parentoni S. Kim T. S. Sanguineti M. C. Phillips R. L. (1998a). Mapping QTLs for ABA concentration in leaves of a maize cross segregating for anthesis date. Maize Genet. Coop. Newslett 72, 72–73.

[B128] Tuberosa R. Sanguineti M. C. Landi P. Salvi S. Casarini E. Conti S. (1998b). RFLP mapping of quantitative trait loci controlling abscisic acid concentration in leaves of drought-stressed maize (*Zea Mays* L.). TAG Theor. Appl. Genet. 97, 744–755. 10.1007/s001220050951

[B129] Unterseer S. Bauer E. Haberer G. Seidel M. Knaak C. Ouzunova M.. (2014). A powerful tool for genome analysis in maize: development and evaluation of the high density 600 k SNP genotyping array. BMC Genomics 15:823. 10.1186/1471-2164-15-823 25266061 PMC4192734

[B130] Van Gioi H. Mallikarjauna M. G. Shikha M. Pooja B. Jha S. K. Dash P. K.. (2017). Variable level of dominance of candidate genes controlling drought functional traits in maize hybrids. Front. Plant Sci. 8:940. 10.3389/fpls.2017.00940 28649253 PMC5465259

[B131] Vincent D. Lapierre C. Pollet B. Cornic G. Negroni L. Zivy M. (2005). Water De cits affect caffeate O-Methyltransferase, Ligni cation, and Related Enzymes In Maize Leaves. a proteomic investigation. Plant Physiol. 137, 949–960. 10.1104/pp.104.050815 15728345 PMC1065396

[B132] Vivek B. S. Krishna G. K. Vengadessan V. Babu R. Zaidi P. H. Kha L. Q.. (2017). Use of genomic estimated breeding values results in rapid genetic gains for drought tolerance in maize. Plant Genome 10, 1–8. 10.3835/plantgenome2016.07.0070 28464061

[B133] Wang R. Yu Y. Zhao J. Shi Y. Song Y. Wang T.. (2008). Population structure and linkage disequilibrium of a mini core set of maize inbred lines in China. Theor. Appl. Genet. 117, 1141–1153. 10.1007/s00122-008-0852-x 18696041

[B134] Wang X. Wang H. Liu S. Ferjani A. Li J. Yan J.. (2016). Genetic variation in ZmVPP1 contributes to drought tolerance in maize seedlings. Nat. Genet. 48, 1233–1241. 10.1038/ng.3636 27526320

[B135] Wen W. Franco J. Chavez-Tovar V. H. Yan J. Taba S. (2012). Genetic characterization of a core set of a tropical maize race Tuxpeño for further use in maize improvement. PLoS ONE 7:e32626. 10.1371/journal.pone.0032626 22412898 PMC3296726

[B136] Witt S. Galicia L. Lisec J. Cairns J. Tiessen A. Araus J. L.. (2012). Metabolic and phenotypic responses of greenhouse-grown maize hybrids to experimentally controlled drought stress. Mol. Plant 5, 401–417. 10.1093/mp/ssr102 22180467

[B137] Wu X. Li Y. Shi Y. Song Y. Wang T. Huang Y.. (2014). Fine genetic characterization of elite maize germplasm using high-throughput SNP genotyping. Theor. Appl. Genet. 127, 621–631. 10.1007/s00122-013-2246-y 24343198

[B138] Xiang Y. Sun X. Gao S. Qin F. Dai M. (2017). Deletion of an endoplasmic reticulum stress response element in a ZmPP2C-A gene facilitates drought tolerance of maize seedlings. Mol. Plant 10, 456–469. 10.1016/j.molp.2016.10.003 27746300

[B139] Xing H.-L. Dong L. Wang Z.-P. Zhang H.-Y. Han C.-Y. Liu B.. (2014). A CRISPR/Cas9 toolkit for multiplex genome editing in plants. BMC Plant Biol. 14:327. 10.1186/s12870-014-0327-y 25432517 PMC4262988

[B140] Xu C. Ren Y. Jian Y. Guo Z. Zhang Y. Xie C.. (2017). Development of a maize 55 K SNP array with improved genome coverage for molecular breeding. Mol. Breed. 37:20. 10.1007/s11032-017-0622-z 28255264 PMC5311085

[B141] Xu J. Yuan Y. Xu Y. Zhang G. Guo X. Wu F.. (2014). Identification of candidate genes for drought tolerance by whole-genome resequencing in maize. BMC Plant Biol. 14:83. 10.1186/1471-2229-14-83 24684805 PMC4021222

[B142] Xu Y. Skinner D. J. Wu H. Palacios-Rojas N. Araus J. L. Yan J.. (2009). Advances in maize genomics and their value for enhancing genetic gains from breeding. Int. J. Plant Genomics 2009:30. 10.1155/2009/957602 19688107 PMC2726335

[B143] Yu J. Holland J. B. McMullen M. D. Buckler E. S. (2008). Genetic design and statistical power of nested association mapping in maize. Genetics 178, 539–551. 10.1534/genetics.107.074245 18202393 PMC2206100

[B144] Zaidi P. H. Seetharam K. Krishna G. Krishnamurthy L. Gajanan S. Babu R.. (2016). Genomic regions associated with root traits under drought stress in tropical maize (*Zea mays* L.). PLoS ONE 11:e0164340. 10.1371/journal.pone.0164340 27768702 PMC5074786

[B145] Zaman-Allah M. Vergara O. Araus J. L. Tarekegne A. Magorokosho C. Zarco-Tejada P. J.. (2015). Unmanned aerial platform-based multi-spectral imaging for field phenotyping of maize. Plant Methods 11:35. 10.1186/s13007-015-0078-2 26106438 PMC4477614

[B146] Zhang G. Guo G. Hu X. Zhang Y. Li Q. Li R.. (2010). Deep RNA sequencing at single base-pair resolution reveals high complexity of the rice transcriptome. Genome Res. 20, 646–654. 10.1101/gr.100677.109 20305017 PMC2860166

[B147] Zhang L. Chia J. M. Kumari S. Stein J. C. Liu Z. Narechania A.. (2009). A genome-wide characterization of microRNA genes in maize. PLoS Genet. 5:e1000716. 10.1371/journal.pgen.1000716 19936050 PMC2773440

[B148] Zhang M. Pan J. Kong X. Zhou Y. Liu Y. Sun L.. (2012). ZmMKK3, a novel maize group B mitogen-activated protein kinase kinase gene, mediates osmotic stress and ABA signal responses. J. Plant Physiol. 169, 1501–1510. 10.1016/j.jplph.2012.06.008 22835533

[B149] Zhang X. Liu X. Zhang D. Tang H. Sun B. Li C.. (2017). Genome-wide identification of gene expression in contrasting maize inbred lines under field drought conditions reveals the significance of transcription factors in drought tolerance. PLoS ONE 12:e0179477. 10.1371/journal.pone.0179477 28700592 PMC5507481

[B150] Zhao F. Zhang D. Zhao Y. Wang W. Yang H. Tai F.. (2016a). The difference of physiological and proteomic changes in maize leaves adaptation to drought, heat, and combined both stresses. Front. Plant Sci. 7:1471. 10.3389/fpls.2016.01471 27833614 PMC5080359

[B151] Zhao Y. Wang Y. Yang H. Wang W. Wu J. Hu X. (2016b). Quantitative proteomic analyses identify aba-related proteins and signal pathways in maize leaves under drought conditions. Front. Plant Sci. 7:1827. 10.3389/fpls.2016.01827 28008332 PMC5143342

[B152] Zheng J. Fu J. Gou M. Huai J. Liu Y. Jian M.. (2010). Genome-wide transcriptome analysis of two maize inbred lines under drought stress. Plant Mol. Biol. 72, 407–421. 10.1007/s11103-009-9579-6 19953304

[B153] Zhu J. K. (2002). Salt and drought stress signal transduction in plants. Annu. Rev. Plant Biol. 53, 247–273. 10.1146/annurev.arplant.53.091401.143329 12221975 PMC3128348

